# Advances in Nanocarrier Systems for Overcoming Formulation Challenges of Curcumin: Current Insights

**DOI:** 10.3390/nano14080672

**Published:** 2024-04-12

**Authors:** Shery Jacob, Fathima Sheik Kather, Mohamed A. Morsy, Sai H. S. Boddu, Mahesh Attimarad, Jigar Shah, Pottathil Shinu, Anroop B. Nair

**Affiliations:** 1 Department of Pharmaceutical Sciences, College of Pharmacy, Gulf Medical University, Ajman 4184, United Arab Emirates; fathima.sheik@gmu.ac.ae; 2 Department of Pharmaceutical Sciences, College of Clinical Pharmacy, King Faisal University, Al-Ahsa 31982, Saudi Arabia; momorsy@kfu.edu.sa (M.A.M.); mattimarad@kfu.edu.sa (M.A.); anair@kfu.edu.sa (A.B.N.); 3 Department of Pharmacology, Faculty of Medicine, Minia University, El-Minia 61511, Egypt; 4 Department of Pharmaceutical Sciences, College of Pharmacy and Health Sciences, Ajman University, Ajman P.O. Box 346, United Arab Emirates; s.boddu@ajman.ac.ae; 5 Center of Medical and Bio-allied Health Sciences Research, Ajman University, Ajman P.O. Box 346, United Arab Emirates; 6 Department of Pharmaceutics, Institute of Pharmacy, Nirma University, Ahmedabad 382481, India; jigar.shah@nirmauni.ac.in; 7 Department of Biomedical Sciences, College of Clinical Pharmacy, King Faisal University, Al-Ahsa 31982, Saudi Arabia; spottathail@kfu.edu.sa

**Keywords:** curcumin, molecular pathways, formulation, nanocarriers, drug delivery, clinical trials

## Abstract

Curcumin, an organic phenolic molecule that is extracted from the rhizomes of *Curcuma longa* Linn, has undergone extensive evaluation for its diverse biological activities in both animals and humans. Despite its favorable characteristics, curcumin encounters various formulation challenges and stability issues that can be effectively addressed through the application of nanotechnology. Nano-based techniques specifically focused on enhancing solubility, bioavailability, and therapeutic efficacy while mitigating toxicity, have been explored for curcumin. This review systematically presents information on the improvement of curcumin’s beneficial properties when incorporated, either individually or in conjunction with other drugs, into diverse nanosystems such as liposomes, nanoemulsions, polymeric micelles, dendrimers, polymeric nanoparticles, solid-lipid nanoparticles, and nanostructured lipid carriers. Additionally, the review examines ongoing clinical trials and recently granted patents, offering a thorough overview of the dynamic landscape in curcumin delivery. Researchers are currently exploring nanocarriers with crucial features such as surface modification, substantial loading capacity, biodegradability, compatibility, and autonomous targeting specificity and selectivity. Nevertheless, the utilization of nanocarriers for curcumin delivery is still in its initial phases, with regulatory approval pending and persistent safety concerns surrounding their use.

## 1. Introduction

Curcumin is a primary constituent found in the rhizome of *Curcuma longa* (L.) and other *Curcuma* spp., commonly known as turmeric, which belongs to the Zingiberaceae family. Turmeric mainly consists of water (80–90%), with carbohydrates accounting for almost 13%, proteins 2%, minerals 2%, and lipids accounting for less than 1% [1]. Curcuminoids, the important compound in turmeric account for 10% of the total dry powder. Curcuminoids primarily consist of curcumin, accompanied by two other diarylheptanoids referred to as desmethoxycurcumin and bis-demethoxycurcumin. The World Health Organization specified a permissible daily intake for curcumin as a food additive up to 3 mg/kg of body weight. Curcumin, being a hydrophobic polyphenol, exhibits a variety of biological and pharmacological effects. Curcuminoids have acquired extensive recognition for their widely reported attributes, encompassing their ability to act as antioxidants, reduce inflammation, and combat microorganisms, in addition to inhibiting the growth of cancer [2,3]. Additionally, research has shown promise in curcumin’s ability to regulate blood sugar levels, offering potential benefits for diabetes management [4]. The potential advantages of curcumin for Alzheimer’s disease [5], atherosclerosis prevention and the management of neoplastic diseases, particularly those affecting the digestive tract, are gaining attention [6]. Its consistent immunomodulatory effects have been demonstrated [7] and have shown notable efficacy in preventing osteoporosis [8], cardiovascular ailments [9], obesity [10] and liver diseases [11]. These findings collectively underscore curcumin’s versatile and promising applications in diverse health conditions. Furthermore, investigations have demonstrated that curcumin influences various cell signaling pathways, enhances the expression of p53, p21, and p27, reduces the levels of gene products promoting cell survival, and triggers apoptosis [12]. Several clinical trials have confirmed its remarkable safety, tolerability, and efficacy, even when administered at elevated oral doses. As a result, it is currently available as a dietary supplement globally [13]. The efficiency of the combined therapy of curcumin with chemotherapeutic agents was reviewed recently [14].

The current review employs a systematic methodology, conducting thorough literature searches across various online databases using key terms such as curcumin, curcumin delivery, nanoparticles, physicochemical properties, molecular pathways, pharmacokinetics, liposomes, nanoemulsions (NEs), polymeric nanoparticles (PNs), polymeric micelles (PMs), dendrimers, solid lipid nanoparticles (SLNs), nanostructured lipid carriers (NLCs), clinical trials, patents, and evaluation. Employing a systematic methodology, the review incorporated structured data extraction, ensuring a methodical and organized approach to gathering pertinent information from diverse sources. The compilation of this information was conducted with precision, weaving together a cohesive narrative that encapsulates the current advancements and emerging trends in curcumin drug delivery. Notably, this approach was tailored to accommodate the distinctive functionalities and intricacies inherent in each database, enhancing the reliability and robustness of the review’s findings.

## 2. Physicochemical Properties

**Figure 1:**
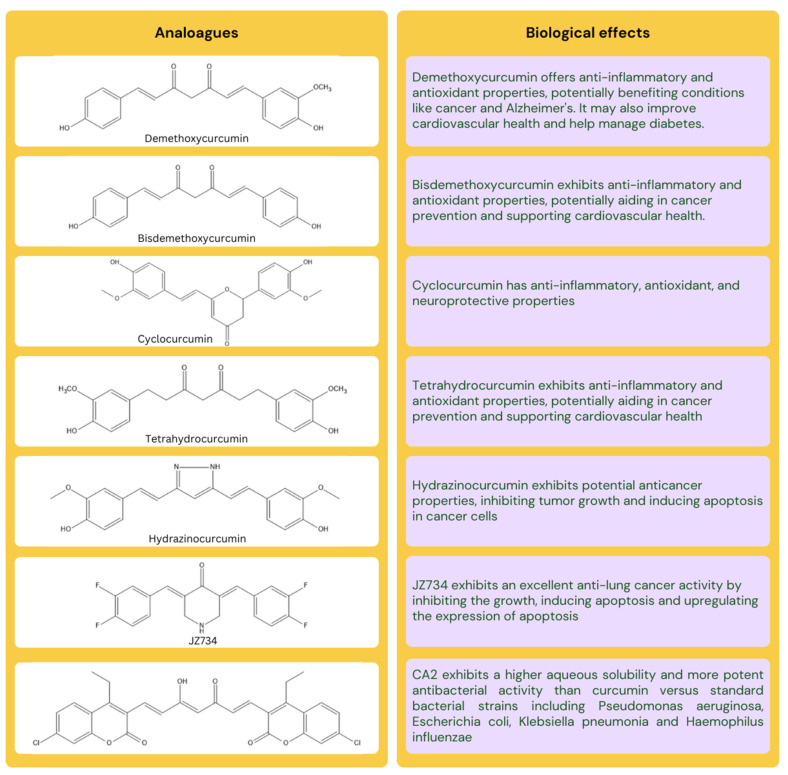
Curcumin analogues and their biological effects.

Curcumin is a polyphenolic compound with the IUPAC name (1E, 6E)-1,7-bis(4-hydroxy-3-methoxyphenyl)-1,6-heptadiene-3,5-dione, and has the chemical formula of C_21_H_20_O_6_. Curcumin consists of three structural fragments: a 7-carbon linker that includes an α, β-unsaturated β-diketone moiety, and two aromatic ring systems with o-methoxy phenolic groups [15]. It possesses hydrophobic characteristics, indicated by a log P of nearly 3.2. This polyphenol exhibits low aqueous solubility (30 nM) under both acidic as well as neutral pH values but demonstrates solubility in semipolar solvents such as ethyl alcohol, methyl alcohol, acetone, alkali hydroxides, glacial acetic acid, and polar aprotic solvents like dimethyl sulfoxide [16]. Its melting point is 183 °C, and its molecular weight is 368.37 g/mol. The enolic and two phenolic protons have been identified as the sources of the three empirically reported pKa values of 10.69 (10.51), 9.30 (9.88), and 8.54 (8.38). The first pKa number represents the dissociation of the enol proton, whereas the values in parenthesis represent the dissociation of the two phenolic protons. Curcumin exhibits numerous methoxy substitutions in its diferuloylmethane chemical structure, resulting in a yellow coloration, particularly within the pH range of 2.5 to 7. Beyond this range, the color transforms into a deep red hue as the pH exceeds the specified limit. Formidable challenges associated with curcumin, including limited bioavailability, insufficient distribution within tissues, rapid clearance, chemical degradation, and toxic potential, have resulted in its categorization as a pan-assay interference compound as well as a failed metabolic panacea molecule [15]. Moreover, turmeric has been found to contain over 50 curcuminoids, including but not limited to bisabocurcumin, curcumalongin, cyclocurcumin, and terpecurcumin [1]. Many reported biological and pharmacological activities can be attributed to the chemical derivatives of curcumin, including demethoxycurcumin, bisdemethoxycurcumin, and cyclocurcumin [17]. Hydrazinocurcumin, a newly developed synthetic analog of curcumin, has demonstrated superior delivery capabilities compared to curcumin and has been extensively researched for its pharmacological effectiveness [18]. The structure of curcumin analogues and their biological effects is illustrated in Figure 1.

## 3. Pharmacokinetics

The reported half-life of curcumin in humans is 6–7 h, posing a significant constraint on its clinical utility [19]. Due to its tautomeric nature, and structural functionalities such as an aromatic o-methoxy phenolic group, alpha, β-unsaturated β-diketone segment as well as a 7 carbon linker, only a limited amount of hydrophobic curcumin is absorbed through the gastrointestinal epithelium [20]. Likewise, in a different clinical investigation, the oral administration of 3.6 g of curcumin produced serum levels of 11.1 nmol/L after 1 h [21]. Additionally, it was found that the intravenous administration of curcumin (10 mg/kg) in rats led to a peak serum level of ~0.36 µg/mL, while a 50-fold dose administration via an oral route resulted in a peak serum level of 0.06 µg/mL [22]. In another study, human volunteers (*n* = 4) ingested 3 g of turmeric, approximately equivalent to 100 mg of curcumin. Plasma evaluation detected curcumin in only one out of four volunteers, reaching C_max_ of 3.2 nM within 2 h after consuming the food. Notably, significant curcumin metabolites, including curcumin glucuronide, demethoxycurcumin glucuronide, and curcumin sulfate, were present in all four human volunteers, with C_max_ values of 47.6 nM, 1.9 nM, and 2.1 nM, respectively, at 30 min after food ingestion [23]. Although curcuminoids demonstrate low oral bioavailability because of their lipophilicity, the unmetabolized compounds can traverse the cerebrovascular barrier. This allows curcumin to reach the brain in concentrations effective for enhancing neuroprotection. The studies carried out in mice administered with an oral dose of 50 mg/kg indicated very low brain concentration after 30, 60, and 120 min. In contrast, an intraperitoneal injection of 100 mg/kg curcumin resulted in concentrations ranging from 4 to 5 µg/g tissue within an average time of 30 min [24]. The reason for reduced levels of curcumin could probably be attributed to the conversion of orally ingested curcumin into water-soluble metabolites, such as glucuronides and sulfates by Phase I and Phase II biotransformation and elimination through the gall bladder. Studies have also demonstrated the conversion of curcumin into dihydrocurcumin and tetrahydrocurcumin [25]. Curcumin undergoes a distinct metabolic pathway facilitated by gastrointestinal microbiota, including *E. coli* and *Blautia* sp. into dihydrocurcumin and subsequently into tetrahydrocurcumin [26]. The limited bioavailability is further aggravated by curcumin binding to enterocyte proteins, potentially altering its structure. In summary, the reduced bioavailability of curcumin can be attributed to several factors such as inadequate gastrointestinal absorption, a high presystemic metabolism rate, rapid elimination, limited penetration, a low targeting capability, susceptibility to alkaline conditions, sensitivity to metal ions, and vulnerability to light besides physicochemical instability.

## 4. Molecular Pathways

**Figure 2:**
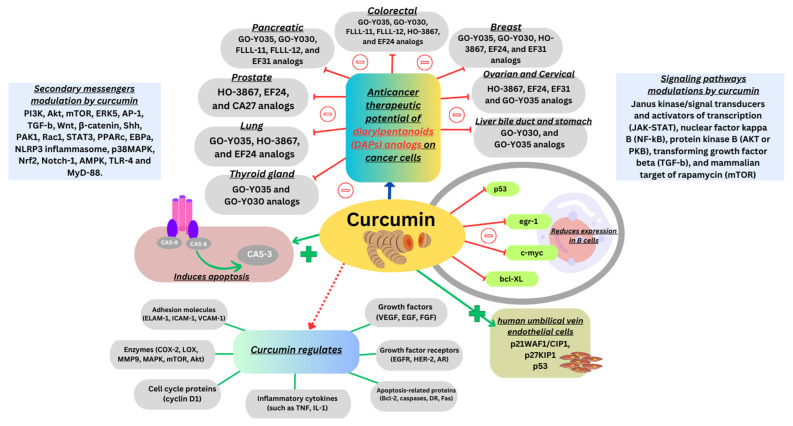
Various molecular pathways and mechanisms of curcumin in cancer therapy.

**Figure 3:**
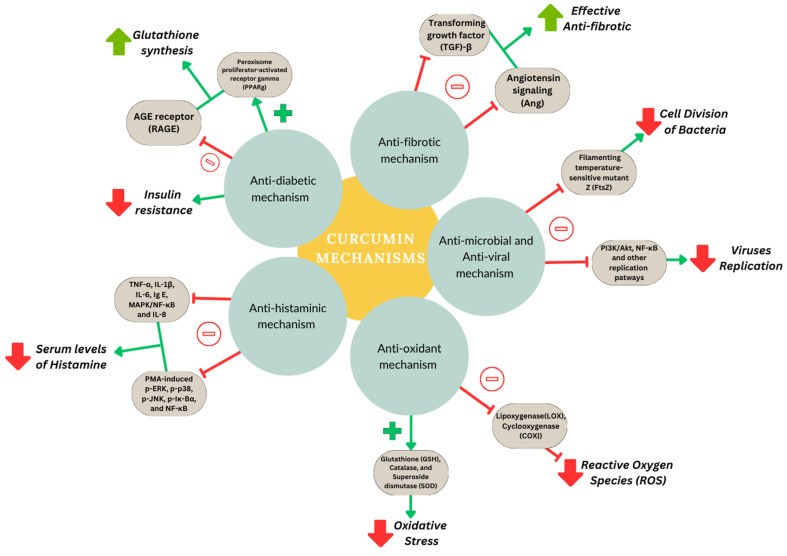
Various molecular pathways and mechanisms of curcumin in noncancer therapy.

Curcumin regulates the expression of inflammatory cytokines growth factors and their receptors, various enzymes, adhesion molecules, apoptosis-related proteins, and cell cycle proteins [27]. Curcumin can also exert its pharmacological effects at its molecular level, by modulating several signaling pathways (Figure 2) [28]. Many studies have demonstrated the significant role of turmeric and its components in preventing or inhibiting cancer. It was found that curcumin reduces the p53 and survival gene expression in B cells (Figure 2). Moreover, curcumin hinders the progression of the cell cycle in endothelial cells present in the human umbilical vein by increasing CDK inhibitor levels (Figure 2). Due to its known capability to interact with multiple targets, curcumin prevents and treats a range of diseases, as described in the introduction section. Figure 2 and Figure 3 display various molecular pathways and the mechanisms of curcumin.

Numerous evidence shows curcumin’s therapeutic potential of 1,5-diaryl-3-oxo-1,4-pentadienes or diarylheptanoids in various cell lines of cancer cells. The compounds exerted an inhibition of cell growth in various cell lines as stipulated in Figure 2 [29]. Curcumin has modulatory effects on several secondary messengers in the cell signal transduction pathway, which are mentioned in Figure 2. Along with reactive oxygen species, curcumin induces Ca^2+^ production, diminishes MMP levels, and enhances caspase-3 activity. It also induces apoptosis further by increasing Bax and decreasing Bcl-2, which releases cytochrome c, thus changing the membrane potential (Figure 2) [30]. In addition, curcumin initiates the increased cleavage of poly (ADP-ribose) polymerase, which again leads to apoptosis [31].

The antidiabetic ability of curcumin is suggested to be due to the production of end products via advanced glycation by the suppressed expression of the respective receptors through the activation of peroxisome proliferator-activated receptor gamma (PPAR-γ) activity, thereby promoting an increase in glutathione (GSH) synthesis. Additionally, the heightened secretion of insulin from pancreatic cells contributes to a reduction in insulin resistance (Figure 3) [4]. Curcumin’s antifibrotic effects are attributed to its ability to hinder the migration, collagen production, and proliferation of fibroblasts by regulating the expression of TGF-β and angiotensin signaling (Figure 3) [32]. The antimicrobial efficacy of curcumin is due to its interaction with FtsZ, a crucial protein responsible for initiating cell division. This interaction disrupts the normal cell division process, thereby inhibiting the growth and proliferation of microbial organisms (Figure 3) [33]. Curcumin demonstrates a remarkable capacity to neutralize free radicals, including oxygen radicals and reactive nitrogen species. Moreover, curcumin can modulate key enzyme activities such as GSH, superoxide dismutase (SOD) and catalase, reinforcing the cellular defense mechanisms against oxidative stress [34]. This compound modulates the metabolism of amyloid beta and exerts inhibitory effects on its aggregation. Additionally, it has an important role in the disaggregation process, i.e., preventing fibrillar amyloid formation, due to its robust anti-amyloidogenic effects [35]. Curcumin’s antiviral properties were demonstrated on viruses of various strains, including feline infectious peritonitis virus, parainfluenza virus type 3, herpes simplex virus, vesicular stomatitis virus, respiratory syncytial virus and flock house virus [36]. Hence, evidence from various sources increasingly suggests that curcumin inhibits viruses and reduces infection through several inhibitory mechanisms. These may mean either directly interfering with the replication of the virus or the suppression of essential viral replication signaling pathways (Figure 3). Investigations revealed that curcumin reduced histamine levels in the serum TNF-α and ovalbumin-specific IgE in allergic rhinitis mice induced with ovalbumin. Along with the suppressive effect on the production of various inflammatory cytokines, this polyphenol considerably inhibited the activation of PMA-induced p-ERK, p-p38, p-JNK, p-Iκ-Bα, and NF-κB. It was disclosed that curcumin modulates mast cell-mediated allergic responses in allergic rhinitis, partly attributed to the suppression of the MAPK/NF-κB pathway [37].

## 5. Role of Nanoparticles in Curcumin Delivery

**Figure 4:**
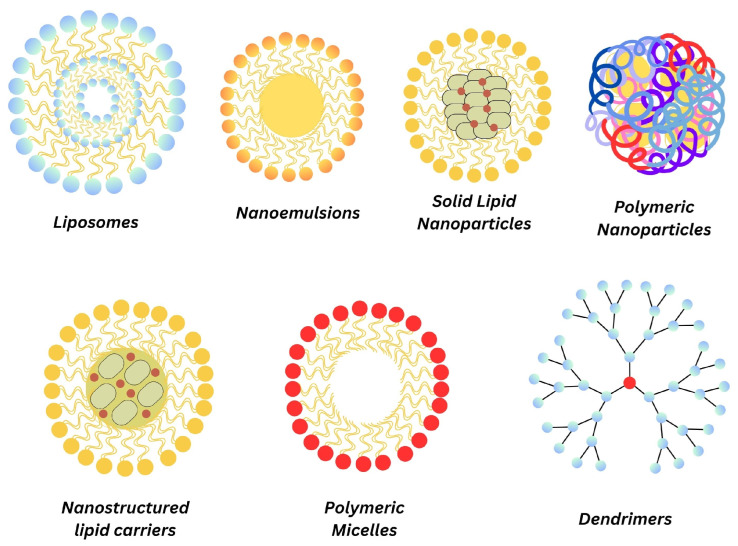
A schematic diagram illustrating various nanocarriers utilized for delivering curcumin.

Despite the fact that curcumin has gained widespread attention for its diverse therapeutic properties, its clinical utilization is hindered by poor solubility, low bioavailability, and rapid degradation in the body. The emergence of nanoparticle-based drug delivery systems shows promise in addressing these challenges and improving the effectiveness of curcumin in diverse medical applications. Nanoparticles can encapsulate curcumin, improving its solubility and protecting it from degradation in the gastrointestinal tract, thereby increasing its absorption and, consequently, enhancing bioavailability. The sustained release profile contributed by nanoparticles can prolong the therapeutic effects of curcumin, reducing the need for frequent administration and hence improving patient adherence. The functionalization of nanoparticles with ligands or antibodies allows for the targeted delivery of curcumin which can maximize therapeutic effects while minimizing side effects on healthy tissues [38]. Furthermore, nanoparticles can facilitate the cellular uptake of curcumin, overcoming barriers imposed by cell membranes. Liposomes, NEs, and lipid nanoparticles such as SLNs and NLCs are commonly used to encapsulate curcumin. Moreover, PMs, dendrimers and PNs comprising biocompatible polymers are utilized to formulate nanoparticles for curcumin delivery, providing sustained release and protection (Figure 4).

### 5.1. Liposomes

Liposomes are lipid bilayer-based tiny vesicles that have the ability to encapsulate medicines, genes, or other bioactive substances. They serve as efficient drug delivery systems, allowing the targeted and controlled release of therapeutic agents, improving the pharmacokinetics and bioavailability of drugs, reducing side effects, and enhancing therapeutic efficacy [39]. Additionally, developments in liposomal technologies have resulted in the creation of multifunctional and stimuli-responsive liposomal formulations, further expanding their applications in diagnostics and personalized medicine [40]. Researchers continue to explore innovative ways to optimize liposomal characteristics and tailor them for specific biomedical applications, reflecting the ongoing evolution and significance of liposomes in modern science and medicine [41]. Liposomes provide several advantages, encompassing high biocompatibility and biodegradability, increased stability, low toxicity, enhanced solubility, targeted cell delivery, controlled distribution, flexibility, and simple preparation [39,42]. A summary of various liposomal curcumin formulations, formulation methods, protocols used, and highlights are described in Table 1. The size was demonstrated to be a pivotal factor influencing the circulation half-life of liposomes, with a direct correlation observed between liposome diameter and their elimination by phagocytes. For instance, phagocytes rapidly eliminate vesicles with the size range of 500 to 5000 nm, while smaller vesicles falling within the range of 20 to 50 nm exhibit lower susceptibility to internalization by macrophages [43]. Furthermore, the number of bilayers impacts the encapsulation quantity of drugs within liposomes. The advancement in liposome technology encompasses the development of various types of liposomes. Stealth liposomes involve coating the external surface with biocompatible polymers like polyethylene glycol (PEG), reducing immunogenicity and macrophage uptake, thereby enabling escape from phagocytosis and increasing biological half-life. However, a drawback is their widespread distribution in tissues. By functionalizing membranes with glycoproteins or ligands for certain receptors, tailored liposomes overcome this restriction and enable preferential accumulation in particular organs for targeted drug delivery. Immunoliposomes functionalized with antibodies aim to deliver entrapped drugs to specific cells, while stimuli-responsive liposomes, such as pH-sensitive or temperature-sensitive liposomes, change in response to environmental conditions for controlled release in desired locations.

Liposomes can function as an effective vehicle to improve the stability, solubility, bioaccessibility, bioavailability, efficacy and targeting properties of curcumin. Numerous studies indicate that liposomes facilitate the solubilization of curcumin within the phospholipid bilayer, enabling its distribution in aqueous mediums and enhancing its overall effectiveness [49]. Chitosan-coated curcumin liposomes were assessed through an in vitro digestion process, enabling the quantification of ingested curcumin’s bioaccessibility [50]. The surface charge turned more positive (+35 mV) after chitosan coating, while the diameter (129 nm) and polydispersity index (PDI, 0.095) stayed the same. Curcumin concentrations in anionic liposomes were marginally greater following the stomach and mouth digesting stages. However, when chitosan-coated liposomes were used as the carrier during the intestinal phase, both in the raw digesta and in the bile salt micellar phase, a higher proportion of curcumin was seen. It has been shown that the existence of a positively charged surface improves curcumin absorption in the small intestine, boosting curcumin’s total bioavailability. Liposomal drugs primarily accumulate in tissues and organs such as the lungs, bone marrow, liver, and spleen [51]. This contributes to enhancing the therapeutic dose range of the drug and reducing side effects. Liposomes have been primarily utilized for delivering anticancer drugs, effectively modifying the biodistribution and clearance of drug molecules. When administered intravenously, liposomes are absorbed by the reticuloendothelial system upon entry into the body.

Quorum sensing is an intercellular gene regulatory system utilized by bacteria to keep an eye on their population and control the expression of virulence genes [52]. How exactly the curcumin liposomes affected the quorum sensing behavior of food-borne pathogens, specifically Aeromonas hydrophila and Serratia grimesii, was investigated. This encompassed factors such as the production of biofilm, extracellular protease activity, swimming motility, and others [53]. The findings showed that the curcumin liposomes had a particle size of around 207 nm, an entrapment efficiency of 82.71%, and a drug-loading rate of 23.33%. It was observed that these liposomes significantly inhibited the quorum sensing systems of the two pathogens, leading to an enhancement in the biological availability of curcumin.

### 5.2. Nanoemulsions

Microemulsions (MEs) are homogenous in appearance, single-phase, clear, and thermodynamically steady colloidal mixtures consisting of water, oil, co-surfactant, and surfactant, with droplets typically ranging up to 100 nm in size. Though a microemulsion has a smaller droplet size compared to Nes, both terms are still being used [54]. MEs present a promising platform for drug delivery, offering improved drug solubility, stability, absorption, and targeted delivery for enhanced therapeutic outcomes. The combined effect of ME-encapsulated curcumin and docosahexaenoic acid in protecting WRL-68 cells from high-fat-induced liver damage and inhibiting LX2 cells has been documented [55]. Co-delivery of these components significantly reduced serum triacylglycerol and low-density lipoprotein cholesterol levels in mice with non-alcoholic fatty liver disease. Pharmacokinetic analysis in rats indicated a higher C_max_ for curcumin, a greater AUC_0–24_, and an extended t_1/2_ for both components.

MEs possess a notable ability to encapsulate hydrophobic agents within their internal oily phase, thereby offering protection against oxidative and enzymatic degradation. In a recent investigation, finely tuned curcumin-encapsulated MEs were developed, comprising 20% geranium oil, 50% Tween 80, and 30% propylene glycol [56]. This formulation exhibited an average droplet diameter of 199.39 ± 0.017 nm, a pH of 4.36 ± 0.057, a conductivity of 40.06 ± 0.05 μS/cm, and a viscosity of 165 ± 0.37 mPa·s. Notably, the ex vivo permeation study demonstrated significant permeation within 24 h, with a higher flux of 130.91 ± 0.02 μg/cm^2^/h. Furthermore, the developed MEs exhibited elevated antioxidant activity and demonstrated superior antimicrobial efficacy against both *E. coli* and Gram-positive *S. aureus* bacteria. Additionally, MEs emerged as a safe and efficient vehicle for curcumin delivery, displaying promising cytotoxic and in vivo anti-inflammatory properties.

NEs have emerged as promising nanocarriers owing to their peculiar ability to entrap both lipophilic and lipophobic moieties, providing an array of options for use in drug delivery. NEs have demonstrated significant potential in enhancing the pharmacokinetics and clinical efficiency of a variety of drugs [57]. The use of medium chain triglyceride as a carrier lipid in emulsions has been demonstrated to significantly enhance the bioaccessibility of curcumin [58]. The application of NE curcumin has been explored for bioaccessibility, wound healing, antiarthritic, anti-inflammatory, antifungal, antiparasitic, quorum sensing, antineoplastic, cardioprotective, and neurodegenerative effects. Table 2 illustrates different preparation methods, oil, surfactant/cosurfactant, biological activities, and key findings of NE curcumin.

The encapsulation of curcumin within NE droplets, composed of oil and utilizing emulsifiers (whey protein concentrate 70 and Tween 80), has been performed [58]. The NE displayed suitable physicochemical properties and stability for oral therapy. Incubation studies in simulated gastrointestinal fluid revealed the NEs’ resistance to pepsin digestion, probably due to the protective role of β-lactoglobulin present in whey protein against pepsin digestion. The overall antioxidant activity of the NE declined marginally due to the encapsulation of curcumin. In brief, the NEs can be regarded as a proficient platform for enhancing the hydrophilicity and bioaccessibility of curcumin, while concurrently safeguarding it from degradation caused by swift hydrolysis and subsequent molecular fragmentation at physiological pH.

Recent research underscores that factors like the nature, category, and amount of the emulsifier play crucial roles in the stability and bioaccessibility of curcumin in NEs [67]. Additionally, interactions between the emulsifier and curcumin are also significant in influencing curcumin delivery by NEs [68]. Sodium alginate showed better stability for curcumin NEs with low concentration (1–1.5%). Nevertheless, this addition led to a decrease in lipid digestibility and drug bioaccessibility [69].

Curcumin exhibits a multifaceted role by enhancing vasoconstriction during the hemostasis stage and scavenging reactive oxygen species, inhibiting NF-κB activity, and reducing TNF-α and IL-1 during the inflammatory phase. In the proliferation and remodeling stages, curcumin facilitates fibroblast migration, collagen deposition, angiogenesis, and transforms fibroblasts into myofibroblasts, contributing to enhanced wound contraction [70,71]. Curcumin supports the wound healing effect by upregulating caveolin-1 expression in epidermal stem cells, where this scaffolding protein contributes to the regulation of cell signaling and membrane dynamics in various cellular processes [72]. Curcumin’s wound healing mechanism was elucidated through its activation of the Wnt signaling pathway and modulation via Dickkopf-related protein 1. Additionally, curcumin exhibited its impact on wound healing by downregulating the expression of MCP-1 by fibroblasts [73]. On the other hand, investigations have revealed a contact-facilitated mechanism wherein NE transports to the intended cell by a contact-facilitated pathway in which it forms a lipid complex and makes surface contact with the outer lipid layer [74].

A curcumin-loaded NE gel for wound healing was developed and evaluated [75]. Among the five prepared NEs (NE1-NE5), NE2 showed a small size (~84 nm), viscosity (~78 cPs) and the highest skin deposition (46.07%). Modifying NE2 into a gel (using 2% chitosan) was found suitable owing to its texture, tactile qualities, and ease of application. The amount of curcumin deposited in the skin by the gel formulation (~980 μg) was greater than NE2 (~771 μg), suggesting the effectiveness of curcumin-entrapped NE gel in wound healing.

In a wound excision rat model, the comparative analysis of healing capabilities of fusidic acid, suspension, clove oil, curcumin-NEs, and control (NE without curcumin) revealed that the wound contraction percentage of curcumin NEs closely approached that of fusidic acid throughout the study period. The study also indicated varying epithelization times for excision wounds: ~15 days for control, ~13 days for clove oil, ~12.5 days for suspension, and ~9.5 days for curcumin NEs and fusidic acid [76]. The permeation capacity of curcumin NEs (~80%) significantly surpassed the suspension (~20%). These research studies provide evidence confirming the high efficiency and effectiveness of NE curcumin in successfully transporting drugs from below the injured skin to the epidermis and dermis.

Natural macromolecule collagen is involved in the differentiation and remodeling stages of wound healing as well as cellular development and proliferation [77]. Investigations reveal how collagen matrices function to be a transport medium for growth promoters, antibiotics, or other therapeutic substances [78]. A study using Wistar rats with excision wounds assessed the efficacy of wound healing with NE curcumin in conjunction with collagen from fish scales and hydroxypropyl methyl cellulose [79]. An ex vivo permeation study of curcumin-loaded fish scale collagen–cellulose-based nanoemulgel displayed a prolonged release (10 h) and lower flux (~12.83 µg/cm^2^/h) compared to other formulations. The results revealed that the prepared nanoemulgel exhibited a complete wound contraction value (100%) in comparison to curcumin NEs (~68%), suspension (~45%), and oral suspension (~35%). Furthermore, the utilization of curcumin nanoemulgel conferred the advantage of being non-sensitive to the skin, as evidenced by a skin irritability grade of <2 (classified as non-irritant), with the absence of significant erythema and edema during 24 h.

To examine the impact of oil globule size on transdermal penetration, curcumin (1%) was incorporated into an oil-in-water NE. Data indicate that NEs with smaller droplet sizes exhibit superior anti-inflammatory activity. This superiority is attributed to the increased likelihood of these small droplets adhering to membranes, covering a larger surface area, and thereby facilitating a more controlled transportation of bioactive NE compounds [80].

Curcumin exhibits potent antibiofilm activity by disrupting or inhibiting quorum sensing, eliminating preformed biofilms, and downregulating virulence factor expression in Aeromonas sobria [81]. A NEs system containing a mixture of catechins, Polyphenon 60, and curcumin was formulated and its combined antibacterial efficacy was evaluated against Escherichia coli [82]. In simulated vaginal media, in vitro drug permeation data revealed that curcumin achieved maximum permeation (~84%) within 5 h, while Polyphenon 60 reached (~91%) within 8 h, with sustained permeation maintained for 12 h. Intravaginal administration of developed NEs in rats resulted in effective dispersion in the site specific regions of the kidney and urinary bladder, which remained active for 12 h compared to the oral and intravaginally administered aqueous solution.

Various investigations have exhibited the efficacy of curcumin against many pathogenic microorganisms, either when used independently or in combination with antibiotics [83,84]. In addition to its known pharmacological safety and efficacy in treating various diseases, curcumin has demonstrated notable antiparasitic effects. In a study targeting Trichinella spiralis adults and larvae in acute and chronic trichinosis models in mice, the efficacy of raw curcumin, chitosan, curcumin-NE, and curcumin-loaded chitosan nanoparticles was evaluated [85]. The findings revealed that albendazole in combination with curcumin NE showed a significant enhancement of efficacy, particularly in adult worms and muscle larvae, leading to an alleviation of pathology in both models.

The effectiveness of a synergistic combination of curcumin and quercetin targeting breast cancer cells (MF-7) was demonstrated [86]. Utilizing a modified emulsification-solvent evaporation method, single NEs of curcumin, quercetin and dual were developed with ideal physicochemical properties. These formulations exhibited moderate hemolysis, indicating biocompatibility when administered intravenously. The IC_50_ was established for quercetin and curcumin, with values of ~40 μM and ~28 μM, respectively. The combination exhibited an IC_50_ value of ~21 μM, suggesting a potentiation effect. A cellular uptake study demonstrated the endocytosis of the drugs within MF-7 cells, with NEs exhibiting approximately a 3.9-fold higher cellular uptake compared to their free quercetin and curcumin (*p* < 0.0001).

Research findings indicate that the antihypertensive efficacy of curcumin NEs is controlled through ACE inhibition, providing cardioprotective benefits against deoxycorticosterone acetate induced hypertension in rats [87]. Curcumin NEs exhibited superior antihypertensive responses when compared to curcumin and the standard drug, captopril, as evidenced by hemodynamic parameters and reduced serum Ang II levels. Furthermore, both curcumin and nanocurcumin elevated levels of GSH, catalase, and SOD, indicating antioxidant effects. Concurrently, they decreased levels of renal function markers, such as serum creatinine and blood urea nitrogen. Notably, curcumin markedly reduced thiobarbituric acid reactive substances levels and histopathological examinations revealed improvements in rats treated with nanocurcumin, underscoring its cardioprotective effects.

NEs loaded with curcumin and quercetin encapsulated utilizing lipoid purified fish oil, castor oil, egg lecithin as the oil phase, and PEG 660 stearate as the surfactant were evaluated for intranasal delivery [88]. Survival tests conducted on the Caenorhabditis elegans Bristol N2 (wild type) animal model revealed the absence of toxicity across all tested formulations and concentrations. The results suggest that the drugs loaded NE may improve nose-to-brain permeability, enabling enhanced treatment efficiency in neurodegenerative diseases.

To optimize drug-delivery properties, it is imperative to extend the circulation of the nanocarrier through PEGylation, thereby ensuring efficient site targeting. The structural influence of the oil phase was revealed by electron paramagnetic resonance spectroscopy, which highlighted curcumin’s stronger stabilizing action in fish oil NE as opposed to its equivalent in soybean oil [89]. Additionally, the experimental setup underscored that, in addition to the oil phase, diverse PEGylated phospholipids and their quantities, coupled with curcumin, collectively exerted a substantial influence on shaping the critical quality attributes of the PEGylated NEs. Conclusively, all products were deemed suitable for parenteral therapy and demonstrated stability over a two-year storage period, as confirmed by preserved antioxidant activity in various assays.

### 5.3. Solid Lipid Nanoparticles

SLNs represent a prospective advancement in drug delivery systems, offering unique advantages in the pharmaceutical field. SLNs utilize solid lipids as a core matrix, creating a stable environment for encapsulating diverse therapeutic agents, thus enhancing drug stability, solubility, and targeted delivery [90]. Furthermore, the adaptability of SLNs distinguishes them as a highly effective nanocarrier system suitable for different types of compounds administered via oral or non-oral routes [91]. The potential of curcumin SLNs has been explored for buccal, oral and parenteral delivery to improve its clinical efficacy in various disorders. Table 3 lists various preparation techniques, brief procedures, results, and key findings of curcumin-loaded SLNs.

To ensure effective buccal delivery, it is crucial to maintain prolonged contact between SLNs and the buccal mucosa. This is essential to mitigate drug loss caused by factors such as continuous salivary flow, mastication, movements of the tongue and lips, and vocalization. To prolong residence time and ensure mucoadhesion, which is vital for local therapy of precancerous lesions, a mucoadhesive poloxamer 407 gel was formulated with curcumin containing SLNs [101]. The data revealed that the gel incorporating drug loaded SLNs demonstrated exceptional mucoadhesion, resulting in an extended (25 min) in vivo residence time. Ex vivo data (utilizing chicken pouch mucosa) indicated enhanced permeation and targeted localization to basal epithelial cells, a favorable outcome for targeting curcumin in malignancies. The curcumin level recovered (in 3 h) from the separated buccal tissue accounts for 21% in SLN gel while it was 2% in control (curcumin in gel). The incorporation of curcumin into SLNs within a gel medium enhanced drug transport into the buccal membranes is attributed to the intrinsic behavior of SLN, which is supported by poloxamer. Erythroplakia patients treated with curcumin-loaded SLNs exhibited a significant reduction in lesion size and pain when compared to individuals who received curcumin gel with no SLNs [101].

The overall bioavailability of bioactive compounds relies on the pathway through which they are delivered into blood, be it via the portal vein or the lymphatic system. The enhanced bioavailability of curcumin can be achieved by directing it to the blood stream via the lymphatic circulatory system, thereby circumventing the biotransformation that takes place in the liver [102]. In addition to enhancing the bioavailability of the compound, transporting curcumin through the lymphatic system has the potential to reduce the spread of tumor metastases by increasing its concentration [103]. SLNs possess the prospective to increase the oral bioavailability of curcumin by effectively immobilizing core materials within the lipid matrix. This safeguarding mechanism protects them against a variety of physical and biochemical factors encountered during transit through the GIT, including pH, ionic concentration, oxidants, and enteric enzymes.

When orally ingested, SLNs with curcumin are broken down by lipases, forming micelles with bile acids, phospholipids, and the resulting solubilized bioactives can then be absorbed into the lymphatic system through enterocytes [104]. It was reported that the oral digestion of SLNs could be modified by adjusting the length and concentration of PEGylated emulsifiers [105]. The lipid portion of the SLN curcumin was generated by mixing Tristearin/canola oil and curcumin in ethanol. Water was used to dissolve PEGylated emulsifiers (PEG10SE or PEG100SE) to create the aqueous phase. The outcomes showed that the micelles made from SLN digesta had a consistent size and surface charge and that over 91% of the curcumin was bioaccessible in all SLNs. The groups treated with PEG100SE stabilized SLNs exhibited the highest C_max_, delayed T_max_ and AUC_0−∞_ compared to others. Indeed, long-PEGylated SLNs demonstrated rapid penetration through the epithelium, because of the micelles’ neutral charge on the surface. This led to a remarkable (12 folds) enhancement in bioavailability in comparison to a curcumin solution in rats. These findings imply that adjusting the interfacial properties of SLNs can significantly impact the bioavailability of curcumin, offering potential avenues for the development of orally bioavailable curcumin formulations.

The oral delivery system’s effectiveness is substantially restricted by the rapid drug release from SLNs in the stomach’s acidic environment. To address this limitation and enhance bioavailability, the surface modification of curcumin-loaded SLN was carried out by coating it with N-carboxymethyl chitosan [106]. The coating leads to a decrease in the rapid release in gastric fluid, showcasing sustained release behavior in intestinal fluid. Moreover, the chitosan coating demonstrated heightened cytotoxicity and improved cell absorption in MCF-7 cells. Surprisingly, developed SLN had a 6.3-fold increase in lymphatic absorption and a 9.5-fold increase in oral bioavailability compared to the curcumin solution.

SLNs have been documented to effectively enhance the pharmacokinetic profile and enable tailored drug distribution to tumor locations, thereby improving efficiency and reducing systemic side effects [107]. Curcumin loaded in SLN and d-α-Tocopheryl PEG 1000 succinate nanocarriers reduced Hodgkin lymphoma xenograft development by 50.5% and 43.0%, respectively, compared to the vehicle treated control; curcumin reduced it by 35.8% when used alone [108]. Additionally, SLN formulation diminished the expression of proteins associated with cell proliferation and apoptosis in lymphoma tumor extracts. In cultured lymphoma cells, curcumin concentration-dependently reduced the expression of pertinent anti-inflammatory cytokines (IL-6 and TNF-α). Moreover, when used in combination with other antineoplastic agents, SLNs exhibited an enhanced growth inhibitory effect.

The phenolic hydroxyl group of curcumin as well as its derivatives play a crucial role in the anti-tumor activity. However, this group is also a major factor contributing to its instability, as it is prone to oxidation, easily degraded by light, and susceptible to color change in an atmosphere that is alkaline [109]. Additionally, curcumin undergoes biotransformation during the body’s metabolism, with its phenolic hydroxyl group interacting with the body’s chemicals like glucuronic acid, sulfuric acid, and glycine, leading to rapid elimination [110]. To enhance the anti-tumor efficacy and stability, efforts have been made to modify the 4-phenolic hydroxyl group within the curcumin structure while retaining the symmetrical phenolic hydroxyl group on the other side [111]. Additionally, utilizing the membrane-sonic approach, derivatives of curcumin (CU 1) were created and added to SLN injection to enhance stability, anti cancer activity, and specificity. Regarding cytotoxicity, the derivative demonstrated ~1.5-fold stronger suppression than pure drug in A549 and SMMC-7721 cells, while derivative-SLN exhibited a 2-fold effect than the derivative. Indeed, both (by 2.6-fold and 12.9-fold, respectively) decreased the toxicity in normal liver cells compared to pure curcumin. In addition, derivative-SLN displayed a substantial apoptotic effect (*p* < 0.05), signifying a potentially effective therapy approach for pulmonary and hepatic cancer.

### 5.4. Nanostructured Lipid Carriers

NLCs, categorized as lipid-based nanoparticles employed in pharmaceutical and biomedical applications, aid as a drug carrier crafted to enhance the solubility, stability, and efficacy of drugs with poor water solubility. NLCs are comprised a combination of solid and liquid lipids, creating a unique nanostructure that enhances the entrapment efficiency and controlled delivery of drugs [112]. The incorporation of both solid and liquid lipids in NLCs allows for a more flexible lipid matrix, minimizing issues such as drug expulsion during storage and providing improved drug-loading capacities [113]. This design offers advantages over traditional lipid-based carriers like SLNs by overcoming limitations associated with stability and offering better control over drug release kinetics. NLCs are employed in various therapeutic areas, including oncology, dermatology, and oral drug delivery. Their versatility makes them appropriate for delivering a large number of drugs, including poorly soluble compounds, and they contribute to advancements in targeted drug delivery and personalized medicine. The utility of NLC curcumin has been evaluated for its topical/dermal delivery by developing different types of gels. Several studies are also carried out to assess the efficiency of NLCs containing curcumin alone or in combination in treating various cancers. Table 4 provides specific details encompassing preparation methods, composition, bioactivity, and highlights of curcumin-loaded NLCs.

NLCs can enhance drug solubility within the matrix and facilitate improved drug transport into and through various layers of skin [121]. Additionally, NLCs are capable of establishing easy contact with the top layer (stratum corneum), leading to a reduction in transepidermal water loss. This reduction in water loss causes more hydration to the top skin section thereby facilitating partitioning and drug diffusion through the skin layers [122]. It was stated that a dual NLC/hydrogel system using Carbopol® (Lubrizol, Rouen, France) as a possible vehicle for topical administration of curcumin to the skin was developed and evaluated [123]. The investigation verified that curcumin antioxidant activity in NLCs was preserved, increasing to a maximum of seven times. Tests for cell viability on fibroblasts and keratinocytes revealed that developed NLCs had modest anti-migration/proliferation effects and non-cytotoxic effects at concentrations as high as 10 μM. Penetration studies indicated greater drug accumulation by NLCs than the NLCs/gels.

Oleogels have been identified as a possible means of delivering bioactive molecules that are hydrophobic [124]. In an attempt to improve curcumin’s stability and entrapment effectiveness, oleogels and NLC have been combined [125]. This combination resulted in superior encapsulation efficiency and manifested a more gradual release of curcumin under acidic conditions, than directly incorporating the drug in the gel. Based on these findings, a dual curcumin delivery system is considered a promising option for specific skin applications, creating opportunities to offer innovative solutions for addressing complex issues in challenging dermal conditions. Moreover, effective control of lipid droplet lipolysis was observed in NLC oleogel developed with carboxymethyl cellulose.

Several studies have explored the therapeutic efficacy of curcumin-loaded NLC in cancer and skin disorders [126,127]. These studies revealed that the formulation’s capacity to maintain product stability, maximize drug loading, and reduce the production of residual solvent contaminants can all be improved by eliminating organic solvents. Moreover, maintaining a particle size below 200 nm could further favor the transport because of its occlusive action. In this regard, research was conducted to develop a topical gel based on NLC that was loaded with curcumin and did not include any organic solvent [128]. The optimized NLCs showed steady drug release for two days, while free curcumin release happened in 4 h. Permeation data signified ~3-fold enhancement in flux and skin retention than control. Cell viability studies indicated no toxicity from the formulation components to keratinocyte cells. Enhanced cell uptake in keratinocyte cells was observed with developed NLC compared to free curcumin dispersion.

Co-loading curcumin along with conventional anticancer drugs in NLCs can lead to enhanced synergistic effects [119]. The selective accumulation of the co-loaded drugs at the tumor site minimizes systemic exposure and reduces off-target side effects, improving the overall safety profile of the treatment. Furthermore, NLCs provide a sustained release of both curcumin and anticancer drugs over an extended period. This sustained release profile can enhance the efficacy by maintaining a therapeutic concentration at the tumor site, thereby preventing rapid drug clearance. Co-loading curcumin with anticancer drugs may help overcome drug resistance, a common challenge in cancer treatment. The multifaceted pharmacological activities of curcumin, including its ability to modulate various cellular pathways, can potentially sensitize cancer cells to conventional treatments, making them more responsive.

NLCs containing docetaxel and curcumin were prepared using glyceryl palmitostearate, trimyristin, medium-chain triglyceride, phospholipon 90GVR, PEG 4000 monostearate stearylamine as lipid components, and solutol HS15VR as the surfactant [129]. Optimized NLCs showed particle size of ~150 nm with a PDI of ~0.263 and ζ potential of ~+26 mV. The % loading for docetaxel and curcumin was ~1.4 and 3, respectively. The formulation exhibited pH independent drug release, with a ~98% drug release at 6 days. The MTT cell viability assay demonstrated significantly enhanced cytotoxicity towards lung carcinoma.

In a clinical trial registered under NCT02439385, the safety and tolerance of a combination therapy involving immunotherapy, bevacizumab, and FOLFIRI chemotherapy were evaluated, alongside the incorporation of ginsenoside-modified NLC with curcumin. According to the study, the long-term survival of patients who received this combination was similar to that of the control group. Notably, the inclusion of curcumin improved chemotherapy compliance, improving the life expectancy for those with metastatic colorectal cancer. Despite limitations due to a small sample size, this study suggests a potential positive association between curcumin and improved patient outcomes in combination with standard chemotherapeutic agents [130].

Using in vitro digestion, a comparative study of several lipid-based nanostructures was carried out to assess the bioaccessibility and possible toxicity of curcumin [131]. The Caco-2 cell line was used to evaluate the cytotoxicity and cellular transit of SLN, NLC, and NE following standardized static in vitro digestion. Curcumin bioaccessibility was highest in NE, NLC, and SLN, with respective values of 71.1%, 63.7%, and 53.3%. Oil-induced cytotoxicity was seen in digested NE and NLC, while non-digested nanostructures and excipients did not exhibit any cytotoxicity. NLC has a higher permeability coefficient than SLN and NE. These findings underscore the significant impact of the physical nature and components of lipid-based nanostructures on their behavior during digestion, as well as their cytotoxicity and intestinal permeability. The study also highlights how crucial it is to perform cytotoxicity analyses following in vitro digestion. The invariable correlation between elevated loading capacity, enhanced controlled release functionality, improved bioavailability of active ingredients, and the final matrix configuration has been emphasized in connection with lipid composition compatibility, the affinity of actives for these elements, and the influence of surfactants [132]. Lipid matrices in SLNs and NLCs have been discussed in relation to one another. Different structural and imaging approaches have been used by numerous investigations in the literature to ascertain the location of solid lipids, liquid lipids, and active components within these matrices [133].

According to a recent study, producing lipid nanostructures requires understanding the effects of different formulation parameters, namely the kind of solid lipid, the amount of liquid lipid, and the kind and number of surfactants. These nanostructures can function either as carriers that are not very soluble in water or as stabilizers in Pickering emulsions. A study was conducted to assess how these product variables impact the globule size, thermodynamic characteristics, entrapment efficiency, drug loading, and storage durability of lipid systems containing the highly hydrophobic active compound curcumin [134]. The initial screening of lipids and investigation of processing conditions aimed to create a suitable lipid structure with remarkably high entrapment and drug loading. It was observed that achieving particle size of desired range (100–200 nm) was crucial for enabling Pickering functionality. As predicted, the polymorphism and crystallinity of the created particles were greatly impacted by the compatibility of the lipid matrix elements as well as the addition of liquid lipid/active chemical, with the latter showing behavior that depended on the liquid lipid concentration. Furthermore, the study reported prolonged storage stability (7 months), indicating the potential practical viability of these lipid systems.

### 5.5. Polymeric Micelles

PMs are nanoscale drug delivery systems composed of amphiphilic block copolymers, which assemble themselves in water to produce core-shell nanoparticles, with the hydrophobic blocks forming the core and the hydrophilic blocks creating the outer shell [135]. The distinctive structure of these particles offers significant benefits, including the enhancement of hydrophobic drug solubility, thereby improving their bioavailability and, consequently, therapeutic efficacy [136]. PMs can facilitate controlled and sustained drug release, resulting in prolonged therapeutic effects and minimized side effects. Biocompatible and biodegradable PMs can be functionalized to facilitate active targeting, allowing for specific drug delivery to target tissues or cells, and contributing to high delivery efficiency. Furthermore, the hydrophilic shell can confer a stealth effect, reducing clearance by the immune system as well as extending bloodstream circulation. PMs can achieve high tumor accumulation through enhanced permeability and retention effects [137]. Additional benefits encompass robust preparation techniques, a remarkable capacity to encapsulate drugs, and great thermodynamic and kinetic stability because of the low critical micellar concentration. The composition, experimental methods, observed activity, and notable highlights of PMs loaded with curcumin are provided in Table 5. The potential of curcumin in various cancer therapies was investigated by developing into PMs as discussed below. Recently, PMs have transitioned to clinical use with the approval of Genexol-PMs (Cynviloq), Paclicals (Apealea), and Nanoxel [138]. Because of such qualities, PMs make great candidates for combinational cancer chemotherapy techniques that attempt to provide synergistic results.

Extensive exploration has been conducted on versatile anticancer delivery systems based on PMs, incorporating stimuli-responsive features and ligand-based targeting. Stimuli-responsive PMs are primarily designed to transport, discharge, and trigger payloads at precise sites, responding to both endogenous and external stimuli, including pH, enzymes, heat, and light. Simultaneously, ligand-modified PMs, utilizing ligands like transferrin, cRGD, EGFR antibody, VEGFR antibody, etc., are predominantly employed to enhance selective internalization into specified tumor cells and areas [138]. A programmable micelle nanosystem, named the dual/redox-sensitive, size-shrinkable, and charge-reversible was designed for the simultaneous delivery of curcumin and NLG919 in chemoimmunotherapy [145]. The outcomes showed that this nanocarrier successfully crossed biological obstacles, resulting in a significant immune response against cancer activation and a reduction in immune resistance. Moreover, it exhibited notable suppression of tumor growth, evasion, and recurrence in animals. The self-assembly of curcumin conjugate with doxorubicin conjugate resulted in mixed micelles [146]. These carriers with a precisely controlled mass ratio of the two drugs, exhibited small particle sizes, a good drug loading ability, and the pH-sensitive release of the drug. The slower drug release leads to a significant reduction in in vivo side effects compared to PMs physically loaded with dual drugs. The synergistic effect was evidenced by their ability to inhibit the development and dissemination of tumors of MDA-MB-231 cells in various assessments, positioning them as viable candidates for safe and efficient cancer combination therapy.

Various amphiphilic polymers, including block copolymers such as poly(2-oxazoline)s, poly(ethylene glycol)-b-poly(ε-caprolactone), and poly(ethylene glycol)-b-poly(lactic-co-glycolic acid, PLGA) were employed for the development of curcumin-encapsulated PMs [147]. A commonly employed strategy to enhance the stability of PMs involves by means of physical interactions between the hydrophobic payload and the hydrophobic core. This encompasses π–π stacking interactions among aromatic groups [148]. Micellar drug delivery systems, formulated with mPEG-b-p(HPMA-Bz) block copolymers, exhibit the potential for scalable production due to particle stability and effective retention facilitated by π–π stacking interactions [149,150]. In another study, PMs encapsulating curcumin were formulated using poly(ethylene glycol)-b-poly(N-2-benzoyloxypropyl methacrylamide), to improve the aqueous solubility and pharmacokinetics of curcumin [151]. The stability of the particles was confirmed as there was no observed alteration in micelle size during 1 day incubation in plasma. In vitro analyses indicated that cancer cells of different types internalized curcumin-loaded micelles cause curcumin-induced cell death. Administering curcumin-loaded micelles labeled with Cy7 via intravenous injection in mice (50 mg of curcumin/kg) revealed a prolonged circulation half-life (42 h) for the micelles. Despite the remarkable solubilizing capabilities of the micelles, they did not induce a cytostatic effect in mice with neuroblastoma, possibly due to the minimum sensitivity of Neuro2A cells to curcumin.

Curcumin exerts its influence on diverse intracellular signaling channels, encompassing enzymes, growth factors, cytokines, and receptors, rendering it a viable option for treating breast cancer [152]. In one attempt involving the 4T1 cancer cell line, PMs of curcumin showed significant anticancer activity. Compared to free curcumin, curcumin micelles had a higher apoptotic score. Additionally, nanosized PMs effectively suppressed the spontaneous pulmonary metastasis of 4T1 cells [153]. Similarly, curcumin PMs formulated with Pluronic demonstrated cytotoxicity against MCF7 cells. The kinetic stability of the micellar solutions was improved by adding the mucoadhesive polymer κ-carrageenan [154].

It was reported that curcumin demonstrates significant protective and therapeutic effects against liver diseases associated with oxidative stress, employing various cellular and molecular mechanisms [11]. These mechanisms involve the suppression of proinflammatory cytokines, lipid peroxidation products, PI3K/Akt, and the activation of hepatic stellate cells. Indeed, curcumin improves cellular responses to oxidative stress by enhancing the expression of Nrf2, SOD, CAT, GSH, GPx, and GR. In brief, curcumin functions as a free radical scavenger, mitigating the activity of various reactive oxygen species through its phenolic, diketone, and methoxy groups. Numerous investigations have consistently shown that curcumin PMs can greatly amplify the compound’s inhibitory effects on HepG2 cell growth [155,156]. Smart nanocarriers, such as stimuli-sensitive PMs, can respond to react to a range of artificial external stimuli as a variety of extracellular and biological stimuli [157]. Given the variation in pH between a solid tumor, blood and normal tissue, pH-sensitive PMs have been engineered to facilitate drug release at the tumor location through the breakdown of pH-sensitive links within the micelle backbone.

The toxicity potential of cationic micelles loaded with curcumin, composed of the triblock copolymer was assessed using cell culture models, including HEPG2 and rat hepatocytes [158]. While HEPG2 cells exhibited no changes in cell viability and membrane integrity, isolated hepatocytes displayed heightened sensitivity to the polymer micelles. Curcumin PMs were investigated for their impact on mitochondrial membrane potential, a crucial indicator of normal mitochondrial function [159]. The researchers observed a noteworthy reduction in the membrane potential when treating HeLa cells with prepared PMs. The findings imply that the apoptosis of HeLa cells might be initiated through the pathway of mitochondrial-mediated apoptosis.

One study explores the possibility of curcumin containing TPGS/F127/P123 mixed PMs as a safe and affordable therapeutic option for cervical cancer [160]. The developed micelles possess ideal pharmaceutical characteristics and demonstrate sustained release over six days. The formulated PMs (NPT100) significantly increase the drug’s selective cellular absorption into HeLa cells instead of NIH3T3 non-cancerous cells. This led to increased cytotoxicity, higher apoptosis, and a significant elevation in the proportion of cells inhibited at the G2/M phase of the cell cycle. Furthermore, PMs demonstrated superior efficacy in activating the mitochondria-mediated apoptosis pathway compared to free curcumin, as evidenced by greater antitumor effects and excellent biocompatibility.

In addition to its anticancer effects on invasion, metastasis, and cell proliferation, curcumin also induces apoptosis and modifies the expression of microRNA [161]. Numerous studies have demonstrated that a variety of molecular targets affect these pathways. One study demonstrated increased apoptosis, a reduction in the expression of the proliferative protein Bcl-2, and an elevation in Bax protein expression by the mPEG-PLA/curcumin PMs on lung cancer cells [162]. Furthermore, curcumin PMs were shown to have the ability to inhibit A-549 cells’ ability to spread by reducing the expression of MMP-2 and MMP-9. The dysregulation of Wnt signaling, a pivotal pathway in carcinogenesis, is well-established in ovarian cancer [163]. Curcumin is acknowledged for its capability in impeding cancer cells from proliferating and growing. It also inhibits the expression and migration of the β-catenin protein through DNA methylation modification. Additionally, this phytoconstituent shows the potential to surmount ovarian cancer’s multidrug resistance [164]. For ovarian cancer, the combined effects of doxorubicin and curcumin enclosed in methoxy poly(ethylene glycol)-poly(L-lactic acid) copolymers were evaluated [165]. Results from the MTT assay and apoptotic study revealed that PMs demonstrated more potent suppression and pro-apoptotic effects on A2780 cells compared to drugs alone. The superior efficacy of PMs in anti-ovarian cancer therapy was confirmed by in vivo tests, wherein the tumor proliferation was inhibited, angiogenesis was suppressed, and apoptosis was promoted.

Curcumin exhibits a significant capacity to inhibit the growth of leukemia cells by causing apoptosis by upregulating the Bax gene [166]. Additionally, it inhibits the expression of genes that are critical for detecting and evaluating the course of leukemia, including FLT3-ITD, WT1, and BCR-ABL, hence suppressing proliferation. Studies on FLT3-overexpressing EoL-1 leukemic cells have shown an enhanced cellular absorption of curcumin-containing micelles [167]. K-562 cells generated from chronic myeloid leukemia and U-266 human multiple myeloma cells were used to evaluate PMs. The results demonstrated improved cytotoxicity against both cell lines and increased cellular uptake, which was ascribed to the interaction between the positively charged micelles and the negatively charged cell surface [168].

The Wnt signaling pathway, which curcumin can potentially impact through upregulation and/or downregulation, plays a crucial role in numerous diseases, including embryonic and organ development [169]. The canonical Wnt pathway stands as a crucial cellular signaling cascade that, through the transcriptional co-activator β-catenin, governs various embryogenic developmental processes and maintains tissue homeostasis [170]. Disruption in the Wnt/β-catenin signaling pathway has been connected to carcinogenesis and contributes to the development of multidrug resistance, as well as the relapse of various tumors [171]. The use of curcumin leads to the decrease of the Wnt/β-catenin pathway, which in turn affects downstream mediators including cyclin D1 and c-Myc. This suppresses chronic inflammation and oxidative stress, exerting control over tumor growth. Moreover, curcumin acts as a PPAR-γ agonist, exerting control over circadian clocks by regulating key circadian genes.

Folate receptors, which are extensively expressed in a variety of malignant tumors, including cells that cause colon cancer, are ligands that folic acid binds to with great affinity. A self-assembled micelle, incorporating folate-modified MPEG-PCL and curcumin, has been devised for colorectal cancer therapy [172]. The developed micelles exhibited a prolonged half-life and greater AUC than the free drug group. The growth inhibitory and pro-apoptotic effects of micelles were shown to be significantly better than those of any other therapy, as demonstrated by the MTT assay and apoptotic investigations. Furthermore, in vivo studies demonstrated that micelles exerted a significantly stronger impact in suppressing tumor growth, promoting tumor apoptosis, and attenuating tumor angiogenesis.

The advantages of pH-sensitive PMs in oral drug delivery include safeguarding the drug from degradation in the upper segment of the gastrointestinal tract, enhancing drug solubilization, and enabling controlled spatial release. In one attempt, researchers developed PM carriers using pH-sensitive N-naphthyl-N, O-succinyl chitosan and N-octyl-N, O-succinyl chitosan to encapsulate curcumin for targeting the colon [173]. The micelle morphology changed in response to varying pH values, indicating pH-responsive characteristics. Curcumin release from the micelles in simulated gastric fluid was restricted to ~20% but exhibited a significant increase in intestinal (~50–55%) and colonic fluid (~60–70%). Remarkably, the naphthyl-based PMs demonstrated the strongest anti-cancer efficacy against the HT-29 colorectal cancer cells.

### 5.6. Dendrimers

Dendrimer nanoconstructs consist of a highly branched star-shaped structure made up of polymeric macromolecules such as polyamidoamine (PAMAM). Certain physical and chemical characteristics of these nanoconstructs include their excellent solubility in water, capacity for encapsulation, monodispersity, and many surface functionalizable groups [174,175].

They are viable options for the administration of both hydrophobic and lipophobic medications due to their capacity to functionalize surface groups. In PAMAM G10, dendrimers have a size of 14.7 nm, compared to 4.3 nm in PAMAM G5. The potential of dendrimers in enhancing the efficacy of curcumin was investigated. The capacity of dendrimers to enhance uptake in cancer cells was assessed across three different types of cancer cells and compared with various nanoformulations of curcumin [176]. The dendrimer formulation exhibited the maximum curcumin uptake into SKBR-3, MDA-MB-231 (breast), and HPAF-II (pancreatic) cancer cells. This was followed by curcumin formulations containing PLGA, β-cyclodextrin, cellulose, and nanogel. The high uptake of curcumin from dendrimers was partly attributed to the positive ζ potential and greater permeation due to the amino groups present in dendrimers. The same study also showed a remarkable binding capacity to plasma proteins and very poor adhesion on red blood cells of dendrimer curcumin nanoconstructs in comparison to other formulations. However, dendrimers showed extensive hemolysis, which can be because of the presence of positively charged surface groups. Therefore, a surface conjugation with PEG would be beneficial in reducing the hemolytic activity of dendrimer formulations. In one attempt, different curcumin derivatives were prepared to examine their potential for both water solubility and cytotoxicity [177]. Among the derivatives tested, the dendrimer-curcumin combination showed higher aqueous solubility as well as cytotoxicity in breast cancer cells, suggesting the potential of the prepared conjugate.

G4 PAMAM dendrimers with amine surface groups are protonated at physiological pH and remain as positively charged amines (NH_3_^+^), which are highly toxic to cells. Replacing the amine groups with neutral hydroxyl groups results in reduced cellular toxicity. In a recently published study, curcumin was loaded into surface-modified PAMAM dendrimers and efficacy was tested in three glioma cell lines [178]. This study concluded that unencapsulated curcumin was ineffective and non-modified dendrimer (G4 NH2) caused substantial death in both normal and cancerous cells, while surface-modified dendrimers showed better activity against glioma cell lines in comparison to control. This investigation clearly indicates the usefulness of surface-modified PAMAM dendrimers as a possible curcumin delivery system in treating glioblastoma.

In addition to increasing curcumin solubility, PAMAM enables surface conjugation of medicines and/or targeted ligands. It was reported that MUC-1 aptamer was attached to curcumin-loaded PAMAM dendrimers that showed high therapeutic index against colorectal cancer adenocarcinoma [179]. Curcumin-loaded PAMAM dendrimers surface modified with triphenylphosphonium ligand for targeted hepatocellular cancer treatment was disclosed [180]. The results indicated that ligand-conjugated curcumin-dendrimer induced apoptosis/cell cycle stoppage at G2/M phase by delivering the drug to the mitochondria of cancer cells. To summarize, PAMAM dendrimers have substantially increased the efficacy of curcumin, especially in treating various types of cancers. Despite the high expense of their preparation, dendrimers can be considered as a potential carrier for curcumin due to their ability to undergo surface modification with ligands for targeted drug delivery.

### 5.7. Polymeric Nanoparticles

PNs present several advantages over other nanocarriers, including a superior gastro-intestinal stability compared to liposomes for safeguarding encapsulated drugs. Additionally, PNs offer various benefits, such as sustained drug release, good storage stability, prolonged blood circulation, and modifiable characteristics [181,182]. The encapsulation of drugs within PNs typically relies on electrostatic forces and/or hydrophobic effects, eliminating the need for chemical modifications. This approach provides the advantage of minimizing the requirement for additional biodegradability and toxicity studies. PNs were tested for their capacity to increase curcumin solubility, drug release, and bioavailability. Furthermore, PN curcumin has been shown to improve endocytosis, microbicidal activity, wound healing, tumor therapy, inflammation, and oxidative stress. A research initiative was undertaken to improve the pharmaceutical and pharmacokinetics properties of curcumin by encapsulating it within PNs composed of carboxymethyl cellulose acetate butyrate [183]. Two precipitation techniques, specifically the conventional and the rapid technique using a multi-inlet vortex mixer were utilized in formulating polymeric matrices loaded with curcumin. The prepared PNs had higher solubility owing to the nano size (150 to 400 nm) and amorphous state of the drug.

Polymers, namely PLGA and polycaprolactone, have gained widespread attention owing to their exceptional biocompatibility and biodegradability, rendering them advantageous for both oral and parenteral drug delivery systems [184]. The mucoadhesive properties of Eudragit nanoparticles make them promising candidates for the oral delivery of curcumin [185]. In one attempt, curcumin-loaded PNs were fabricated using the above-mentioned polymers and compared various properties. The particles prepared with Eudragit showed a smaller size, superior redispersibility post freeze-drying and rapid release (90% in 1 h), compared to other PNs tested [186]. There is another study that reported a higher endocytosis of curcumin by the colon adenocarcinoma cell line (HT29 cells) when encapsulated in PLGA PNs, as opposed to free curcumin [187]. These PNs exhibited outstanding colloidal stability in simulated gastrointestinal fluids and maintained excellent long-term storage stability.

Despite the utilization of curcumin as a photosensitizer, its real time application is limited due to challenges such as low water solubility, instability, and diminished bioavailability [188]. Attempts were made to develop cationic and anionic PNs of curcumin by nanoprecipitation using polylactic acid and dextran sulfate [189]. A comparative study was conducted between PNs and free curcumin against planktonic and biofilm cultures. Anionic PNs demonstrated a decrease in the photoinactivation of biofilms, whereas the cationic PNs exhibited a microbicidal effect without the presence of light. Anionic PNs did not show cytotoxic effects in comparison to others. The study confirms a stronger antimicrobial photodynamic impact on planktonic cultures compared to biofilms, and the entrapment of the drug in anionic PNs mitigated its cytotoxicity.

Natural polysaccharides, such as chitosan, have been utilized for the treatment of wounds and burns due to their hemostatic properties, ability to stimulate healing, and antimicrobial effects. On the other hand, synthetic polymers like polyvinyl alcohol have demonstrated wound healing properties in various investigations, while PLGA has been identified to support angiogenesis through lactate supply, thereby facilitating the wound healing process [190]. Additionally, semi-synthetic polymers like carboxymethyl cellulose improve the solubility of curcumin and demonstrate wound healing properties [183]. Comparative data of nanoparticles containing curcumin prepared using these polymers showed entrapment efficiency decreases as; PLGA > chitosan > cellulose [191]. However, the chitosan-based nanoparticles expedited the wound healing process, attributed to the synergistic effect of curcumin and chitosan.

Multi-drug chemotherapy has emerged as a widely adopted strategy for treating malignant tumors, demonstrating commendable therapeutic outcomes. A recent study explored the simultaneous administration of paclitaxel and curcumin PNs to exert an antitumor effect against breast cancer [192]. These PNs demonstrated cytotoxicity in MCF-7 cells in a dose-dependent manner, resulting in a higher apoptosis rate (~64%) compared to free drugs (~34%). Anti tumor effect of PNs in BALB/c nude mice xenografted with MCF-7 cells revealed a substantial suppression of tumor growth, extended survival time, and decreased side effects than free drugs. Additionally, the PNs resulted in decreased Ki67 expression and increased TUNEL positivity, indicating enhanced apoptosis in tumor cells in comparison to other groups, highlighting the beneficial effect of PNs.

PNs containing curcumin are suggested to be suitable for treating diseases linked to oxidative stress and inflammation. The polymeric matrices were developed using N-vinyl caprolactam, 1-vinyl-2-pyrrolidone, and a bioactive terpolymer based on α-tocopheryl methacrylate [193]. Human articular chondrocyte and RAW 264.7 cultures were subjected to cellular assays to assess the compounds’ cytotoxicity, cellular uptake, antioxidant, and anti-inflammatory properties. The systems demonstrated antioxidant activity through the DPPH assay and measuring cellular reactive oxygen species. In chondrocytes, the systems reduced pro-inflammatory factors such as IL-8, MCP, and MIP, while in RAW 264.7 they decreased nitric oxide, IL-6, TNF-α, and MCP-1, showcasing anti-inflammatory potential. Moreover, biocompatibility was validated in rats by administering subcutaneously. In another attempt, poly-glycerol-malic acid-dodecanedioic acid polymer containing curcumin nanoparticles was formulated [194]. The activity was evaluated in various breast cancer cell lines. The data revealed apoptotic features and nuclear anomalies in the treated cells which was confirmed by the overexpression of caspase 9.

A copolymer nanoparticle formulation containing curcumin has been investigated in the management of liver inflammation [195]. The results revealed a significant reduction in liver inflammation in diabetic animals, as indicated by elevated oxidative stress markers (hepatic MDA and NO), decreased GSH levels, alongside alterations in other biomarkers. Moreover, diabetes substantially increased the serum concentration of NF-ҡB, hepatic COX-2, and TGF-β1, concurrently reducing hepatic PPAR-γ. The findings indicated that hybrid nanoparticles were more effective than their free counterparts.

## 6. Clinical Translation and Future Perspectives

Several preclinical studies have indicated significant advantages in cancer treatment through the combined use of curcumin and traditional medications. To evaluate potential pharmacokinetic interactions between concurrently administered curcumin, often referred to as dietary supplements and chemotherapeutic agents, clinical trials were conducted across various cancer types [196]. In a clinical trial with multiple phases that included participants with colorectal metastases, a commercially standardized extract of curcumin (C3 complex, Sabinsa Corporation, East Windsor, NJ, USA) was incorporated alongside FOLFOX, which is a conventional chemotherapy regimen centered around oxaliplatin [197]. To evaluate the advantages of curcumin to surmount the adverse effects induced by FOLFOX and/or the influence on patients well being, a subsequent phase II trial was carried out. Data analysis interpreted from this trial suggests that combining curcumin with FOLFOX chemotherapy is both safe and well-tolerated, showcasing potential benefits for cancer patients (NCT01490996). Curcumin possesses the ability to inhibit the metabolizing enzyme UDP-glucuronyltransferases, suggesting its potential to stabilize the vital metabolite (SN-38) of irinotecan and influence its pharmacokinetics. In a dose-escalation investigation, the curcumin–phosphatidylcholine complex (Meriva) was examined in combination with a consistent dose of irinotecan, selected for its enhanced absorption in comparison to alternative curcumin formulations [198]. The pharmacokinetic assessment revealed that curcumin did not cause any changes in the pharmacokinetics of irinotecan (NCT01859858). Table 6 provides a summary of clinical trials, related to various curcumin-loaded nanocarriers designed for delivery. Researchers are still exploring the diverse biological activities of curcuminoids, aiming to harness their potential for promoting overall health and addressing various health conditions. Table 7 provides recent instances of patented curcuminoids, accompanied by a brief overview of their innovative features.

A significant hurdle associated with the formulation development of curcumin is its limited bioavailability and susceptibility to light, heat, and oxygen. This vulnerability contributes to stability concerns during both storage and administration. Nanocarriers can help enhance the solubility and stability of curcumin, increasing its absorption and bioavailability in the body. Functionalized nanocarriers can be designed to transport curcumin directly to the desired tissues or cells, increasing therapeutic efficacy while minimizing side effects. This targeted delivery is particularly valuable in tumor therapy and inflammatory diseases. Curcumin can be co-delivered with other therapeutic agents or drugs within nanocarriers to create synergistic effects, enhancing the overall therapeutic outcome [14]. The combination of curcumin with other drugs in a co-delivery system presents several advantages, including decreased drug resistance, improved bioavailability, and enhanced bioactivity through the targeted localization of curcumin. This approach may be explored for various diseases, including cancer and neurodegenerative disorders. The biocompatibility and potential toxicity of nanocarriers need thorough investigation, especially since certain materials used in nanocarrier formulations may raise concerns about their safety in clinical applications. Transitioning from laboratory-scale synthesis to a large-scale production of curcumin-based nanocarriers poses challenges in terms of scalability, reproducibility, and cost-effectiveness. Despite promising preclinical studies, translating curcumin-based nanocarriers into clinically effective therapies requires rigorous clinical trials to establish safety, efficacy, and optimal dosages. Achieving consistent therapeutic outcomes in diverse patient populations remains a challenge since patient responses to curcumin-based nanocarriers may vary due to factors like individual variations in metabolism, disease stage, and genetic factors. Conjugating antibodies or ligands with nanocurcumin can overcome their limitations, potentially serving as an excellent drug candidate.

## 7. Conclusions

The functional properties of curcumin have been significantly boosted through innovative nano-sized strategies and codrug delivery approaches. These advantages, coupled with curcumin’s minimum toxicity, suggest the ability of nanocurcumin as a promising alternative for enhancing its transport to diverse target organs. The performance of the nanosystem relies on judiciously choosing the materials employed, the delivery route, and the administered dose.

## Figures and Tables

**Table 1:** Preparation methods, procedure, and important findings of curcumin encapsulated liposomes.

Methods	Procedure	Highlights	Reference
Thin film hydration	Phospholipid and curcumin were initially dissolved in absolute ethanol, followed by the removal of ethanol through rotary evaporation. The formed film was hydrated with ultrapure water for 30 min to obtain curcumin-loaded liposomes.	The diffraction pattern of thin film curcumin showed a crystalline structure. Liposomes were unstable during storage (4 °C for 1 month) and exhibited leakage of curcumin. The transformation levels of the formulation were 43.4%, and the bioaccessibility was 55.4%, similar to the pH-driven method (54.1%).	[44]
Thin film hydration	Soybean lecithin, cholesterol, DSPE–MPEG 2000, curcumin, and tetrandrine were dissolved in methanol and chloroform in a round-bottom flask and evaporated slowly leading to the formation of yellowish clear film. Ultrapure water was added to the blend and subjected to ultrasonication, followed by extrusion and filtration to remove any remaining drugs. The resulting liposomes were then transformed into nanoliposomes.	Nanoliposomes showed nanosized (<100 nm) vesicles, high drug entrapment capacity, loading efficiency and release characteristics. In vivo studies demonstrated the formulation has no significant toxicity on zebrafish. The tumor cytotoxicity test confirmed that nanoliposomes had a strong inhibitory effect on a variety of cancer cells. Furthermore, it enhanced the physical and chemical characteristics of both drugs, rendering them safer and more effective.	[45]
Thin film hydration	A round-bottom flask containing cholesterol (50 mg), Tween 80 (50 mg), and curcumin (1 mg) was dissolved in chloroform (10 mL) and evaporated (using Rotavapor at 50 °C) to develop a thin film. The thin film was hydrated with water (10 mL) and the flask was subjected to manual shaking (30 min) and sonication (3 min) using a bath sonicator.	Formulated curcumin carriers exhibited size, zeta potential and encapsulation efficiency of ~271.3, −61.0 mV, and 81.1%, respectively. Both concentrations of curcumin-loaded liposomes (1 and 5 μg/mL) demonstrated a substantial (*p* < 0.05) decrease in the expression levels of pro-inflammatory markers (IL-6, IL-8, IL-1β, and TNF-α) against the positive control group. Liposomal curcumin at 1μg/mL exhibited a more pronounced reduction in inflammatory markers compared to the concentration of 5 μg/mL.	[46]
Thin film hydration with hand extrusion	Lipid films were generated via rotary evaporation and hydrated with HEPES-buffered saline. Formed liposomes underwent 41 passages through an 80 nm polycarbonate membrane during extrusion. Non-incorporated crystalline or aggregated curcumin was eliminated through centrifugation for 15 min.	The findings indicate that the inclusion of the fluid phospholipid dioleoylphosphatidylcholine in liposomes enhanced the aqueous solubility and stability of curcumin. The in vivo effectiveness of curcumin achieved through reduced decomposition and accumulation in tumor tissue was hindered by the premature release of curcumin. Cytotoxicity and uptake experiments distinctly demonstrated a diminished effectiveness of curcumin liposomes.	[47]
pH driven	Ultrapure water, curcumin, and phospholipid were combined and stirred for a minimum of 4 h at room temperature. Subsequently, the solution’s pH was raised to 12 using 4 M NaOH. After 20 min of stirring, the pH was fixed at 5.3 with 4 M HCl, and the resulting pH-driven curcumin liposomes were collected.	Under alkaline conditions (pH 12), the mean vesicle size and zeta potential of the liposomes reduced from 980.5 to 220.3 nm and −53.7 to −91.9 mV, and the drug loading was ~3.0%, respectively. At acidic pH (5.3), encapsulation efficiency, average particle size, polydispersity index, and the zeta potential of the liposomes were measured as approximately ~62.8%, 217.5 nm, 0.248, and −53.1 mV, respectively. Microscopy images revealed spherical and multilamellar vesicle structures. The formulation demonstrated high bioaccessibility and sustained stability throughout storage.	[44]
Freeze-drying	The combination of the dispersions curcumin–hyaluronan in water and curcumin–Eudragit S100 in ethanol with soy phosphatidylcholine (Phospholipon^®^ 90, P90G, Pfizer, New York, NY, USA) using sonication resulted in the formation of Eudragit–hyaluronan liposomes. Similarly, Eudragit-nutriosomes were produced by mixing dispersions of curcumin–Eudragit S100 (Evonik Industries AG, Darmstadt, Germany) in ethanol and curcumin–Nutriose FM06 (Roquette, Lestrem, France) in water with soy phosphatidylcholine using sonication. The resultant curcumin-loaded liposome vesicles underwent freezing (−80 °C) and freeze-drying 24 h at 90 °C at 0 mm Hg. Samples were then rehydrated with water and sonicated to obtain a curcumin concentration of 10 mg/mL.	The antimalarial efficacy of orally administered curcumin encapsulated in Eudragit-nutriosomes was demonstrated in Plasmodium yoelii-infected mice. The investigation revealed that highly stable nutriosomes, consisting of P90G (160 mg/mL), curcumin (10 mg/mL), and Nutriose (50 mg/mL), improved the accumulation of curcumin in the intestines and facilitated absorption to enhance curcumin bioavailability.	[48]

**Table 2:** Preparation method, formulation components, biological activity and highlights of curcumin-loaded nanoemulsions.

Methods	Surfactant, Co-Surfactant and Oil	Biological Action	Key Findings	Reference
High-pressure homogenization	Solutol-HS 15, soyabean oil	Antiarthritic activity	Curcumin NEs reduced NF-κB expression and inhibited the release of inflammatory facilitators, including TNF-α and IL-1β. An optimized formulation demonstrated that the AUC and C_max_ were more than three-fold greater than those obtained with the nanosuspension, indicating improved bioavailability in rats. Both intravenous injection and oral delivery of nanoformulations yielded comparable therapeutic effects in rheumatoid arthritis.	[59]
Hot homogenization technique	Sodium oleate, lecithin, palm oil, medium chain coconut oil	Anti-inflammatory activity	Curcumin NEs administered at doses of 20 and 40 mg/kg in male rats with carrageenan-induced paw edema resulted in a 33% and 56% inhibition of paw edema, respectively, at 5 h. Notably, the inhibition of edema volume by the 40 mg/kg dose of curcumin nanoemulsion was comparable with the standard drug ketorolac at a dose of 2.7 mg/kg.	[60]
Interfacial pre-polymer deposition and spontaneous nano-emulsification	Medium chain-triglyceride, soy phospholipids, Poloxamer 188	Antineoplastic activity	Curcumin NEs can be considered a promising therapeutic option for oral squamous cell carcinoma, potentially inhibiting cell proliferation via the downregulation of PI3K/Akt/mTOR pathway and the upregulation of miR-199a. CUR-NEs effectively counteract the impact of a miR-199a inhibitor on the cell proliferation of carcinoma cells and the phases of cell cycle multiplication in a timely manner.	[61]
Self-micro emulsification	Cremophor RH40, glycerol, medium chain triglyceride	Antineoplastic activity	Particle size, zeta potential, and PDI values for curcumin NEs were ~35 nm, –8.54 mV, and 0.132, respectively. The formulation exhibited significant inhibitory effects on the proliferation of PC-3 cells in a dose- and time-dependent manner, particularly at concentrations exceeding 20 µmol/L. MTT assay results indicated heightened cell cytotoxicity, likely due to increased cellular apoptosis and G2/M phase cell cycle arrest. Recorded 1.4- and 1.7-fold increases in Peff for nanoemulsion in the duodenum (1.80 × 10^−3^) and jejunum (1.59 × 10^−3^) compared to the free drug indicates the possibility of improving the oral delivery.	[62]
Self-nano emulsification	Glyceryl monooleate, PEG 5000, cremophor EL 100	Antifungal activity	The average droplet size, ZP, and PDI of curcumin NEs were ~90 nm, −7.4, and 0.171 mV, respectively. The zone of inhibition against Candida albicans by nanoemulsion (~24 mm) and nanogels (~30 mm) were significantly larger than those of marketed Itrostred gel (~22 mm).	[63]
Spontaneous emulsification	Tween 80/Tween 85, ethanol, soyabean oil	Antimicrobial activity	The mean particle diameter and zeta potential of formulation were found to be ~60 nm and −16 mV, respectively. At concentrations of 1250 and 625 μg/mL, a 60-min exposure resulted in mortality rates of 94% and 73.33%, respectively, and complete mortality was observed after 120 min. Differential interference contrast microscopy revealed extensive alterations in the tegumental surface of exposed protoscoleces, suggesting that curcumin NEs could serve as effective and low-toxicity protoscolicidal agents.	[64]
Spontaneous emulsification	Tween 80, castor oil, soy lecithin	Protection against intestinal damage	Optimized curcumin NEs showed a size of 409.8 nm, PDI of 0.132, and zeta potential of −18.8 mV. The experimental trials with mice did not show a significant reduction in inflammation in the intestinal injury caused by indomethacin due to gastric instability. But, the group treated with NEs exhibited a higher relative abundance of the genus Lactobacillus (*p* < 0.05) and hence has relevance in the modulation of the intestinal microbiota.	[65]
Spontaneous emulsification	Tween 80, Labrafac PG	Spermatogenesis	Per the oral administration of curcumin NEs at 5 or 10 mg/kg to rats, notably improved spermatozoa motility, restored amino acid balance in semen, normalized serum leptin and testosterone levels were observed, and oxidative and nitrosative parameters were brought to normal levels compared to curcumin powder. Furthermore, reduced testicular DNA fragmentation and increased testicular cellular energy was observed. In addition, curcumin NE (10 mg/kg) mitigated the adverse effects of a high-fat, high-fructose diet on spermatogenesis.	[66]

**Table 3:** List depicting various preparation techniques, brief procedure, results and key findings of curcumin-loaded solid lipid nanoparticles (SLNs).

Technique	Procedure	Results	Key Findings	Reference
Emulsification and low-temperature solidification	To create the organic phase, curcumin, stearic acid, and lecithin were dissolved in chloroform. Simultaneously, the aqueous phase was formed by dissolving Myrj52 in distilled water. The combined phases were agitated (1000 rpm) for a period of 1 h at 75 °C. After centrifugation, the resulting mixture was reconstituted in pure water, frozen at −80 °C for 24 h, and then subjected to lyophilization.	Curcumin-SLNs exhibited a distinct spherical shape, measuring approximately 40 nm and possessing an anionic surface potential. The drug payload and entrapment capacity in SLNs achieved 23.38% and 72.47%, respectively. Western blot analysis revealed an elevation in the Bax/Bcl-2 ratio, accompanied by a decrease in the expression of cyclin D1 and CDK4.	Developed SLNs exhibited improved efficacy on SKBR3 cells. The optimized batch, with a small size (~30 nm) and negative charge, resulted in significant cell death rate and promoted apoptosis, compared to plain curcumin. In addition, SLNs exhibited the capability to inhibit cell migration.	[92]
Emulsification–ultrasonication	The process involves the melting of glyceryl monostearate with curcumin at 80 °C, while the aqueous phase comprises Tween 80. The two phases are then mixed and blended using a high-speed homogenizer (5000 rpm), followed by ultrasonication with a probe sonicator to form an oil-in-water nanoemulsion. It is rapidly cooled in an ice water bath, leading to crystallization and the formation of SLNs.	Optimized SLNs have low particle size (115 nm), a PDI of ~0.112, a ZP of ~−32.3, an mV; EE of ~70%, and a DL of ~0.81%. Prepared SLNs exhibited a 99.32% drug release for 120 h. MTT assay demonstrated that after 48 h incubation, the formulation exhibited increased cytotoxicity with an IC_50_ value of ~26.12 µM, more than free curcumin (IC_50_ of ~35.12 µM). Endocytosis of curcumin was greater with SLNs (~682 ng/µg) in comparison to free curcumin (~162 ng/µg).	Curcumin SLNs exhibited all the desirable features of nanoparticles. TEM micrographs displayed a spherical shape and uniform surface texture. The formulation displayed a sustained release pattern over 120 h. The loading of curcumin into the SLNs notably enhanced uptake by A549 cells.	[93]
High pressure homogenization	The aqueous phase, comprising Tween 80 and curcumin, was kept at room temperature. The oil phase, containing cholesterol, was dissolved in a mixture of ethanol and acetone (3:1) and maintained at 75–80 °C. The hot oily phase was then mixed with the aqueous phase under homogenization (11,000 rpm for 7 min). The resulting mixture was gradually cooled to room temperature to obtain SLNs. The SLNs were lyophilized using mannitol as a cryoprotectant to enhance stability.	Before freeze-drying, particle sizes were 112 nm with a PDI of 0.114, and the utilization of 5% and 15% mannitol as cryoprotectants led to larger particle sizes of 163 nm and 306 nm, respectively. SEM demonstrated spherical particles. Before and after freeze drying loading efficiency was ~70%, and over 85%, 92% of the loaded curcumin was released after 36 and 48 h, respectively. SLNs exhibited an enhanced antimicrobial effect against both *E. coli* and *S. aureus.*	The freeze-drying process can yield desirable characteristics such as particle size, drug entrapment, and extended-release pattern. SLNs have the potential to decrease the concentration of curcumin needed to inhibit bactericidal activity.	[94]
High pressure homogenization	The aqueous phase consisted of Tween 80, phospholipon 90G, and water maintained at 80 °C. Curcumin, dissolved in PEG 400, was added to the melted lipid phase consisting of Compritol®888 ATO (Gattefossé India Pvt. Ltd, Mumbai, India) and GMS (4:1). The lipid mixture was then added to the aqueous phase with high-speed homogenization (8000 rpm for 8 min) and further processed through 3 cycles at 500 psi. Dispersion was cooled to room temperature to obtain SLNs.	The amorphous nature of SLNs was indicated by PXRD and spherical nature was confirmed by field emission scanning microscopy. Curcumin-loaded SLNs were released for a duration up to 120 h, achieving a release of 99.73 ± 1.12% and exhibiting zero-order release kinetics. In contrast, free curcumin exhibited a first-order release, with complete drug release occurring within 24 h.	SLNs exhibited a remarkable curcumin drug loading of 15 mg/mL, confirming the amorphous state of curcumin within the SLN. The controlled-release nature of SLNs was indicated by a zero-order release pattern. Photodecomposition studies proved the stability of curcumin-within SLNs.	[95]
Homogenization and ultrasonication	Curcumin was included in the melted cetyl palmitate while stirring continuously. The lipid phase underwent a dropwise addition of an aqueous solution containing Tween 80 under magnetic stirring, followed by a 5 min ultrasonication at 30% amplitude. The resulting lipidic dispersion was subsequently cooled to room temperature to yield SLNs loaded with curcumin.	The study revealed that increasing the % of oleic acid significantly reduced the particle size of SLNs. The optimized SLNs exhibited a particle diameter of ~204 nm and a PDI of ~0.194. The amorphous nature of the drug within the lipid matrices is confirmed by DSC and XRD. Antioxidant activity showed no difference between free curcumin and SLNs. Cells treated with SLNs demonstrated high viability at lower concentrations (1 and 10 μg/mL). The prolonged retention time in plasma and increased half-life of curcumin in SLNs showed sustained release.	The pharmacokinetic investigation in rats demonstrated that amount of curcumin present in the brain was predominant (*p* < 0.005) in SLNs (AUC_0–t_, 116.31 ng/g.h) in comparison to plain curcumin. Free radical scavenging study using DPPH assay revealed that preparation processes do not exert any impact on the anti-oxidant activity of curcumin.	[96]
Hot homogenization method combined with ultrasonication	Curcumin was dissolved in the melted lipid phase of Compritol^®^ 888 ATO. It was combined with the Poloxamer 188/Transcutol P dissolved in hot aqueous solution to form O/W primary emulsion. It was further homogenized, sonicated and cooled to room temperature to generate SLNs. Compritol^®^ 888 (4%, 5%, and 6% *w*/*w*), Poloxamer 188 (2.2% to 3.3% *w*/*w*) and Transcutol (2% or 4%) were incorporated as inactive ingredients.	SLNs demonstrated the capacity to encapsulate a substantial amount of the drug with an encapsulation efficiency percentage ranging from 97% to 99%. Curcumin loading into the lipid phase of SLNs significantly enhanced cellular uptake by A549 cells. SLNs maintained stability at 25 °C/60% relative humidity for the entire 3-month study period.	The inclusion of Transcutol in SLNs enhanced their ability to interact with cells. Empty- Transcutol 4%-SLN 4% and, curcumin- Transcutol 4%-SLN 4% demonstrated the capability to alter the lipid profile and metabolism of 3T3 fibroblast cells as a result of SLN uptake.	[97]
Hot homogenization technique followed by ultrasonication	Melt and blend a mixture of solid lipids, consisting of γ-aminobutyric acid and γ-amino-α-hydroxy butyric acid, with curcumin in a hot water bath (85–90 °C), while a surfactive agent or surfactant combination is solubilized in water at the same temperature. The hot surfactant solution is gradually introduced to the lipid melt (85–90 °C) and stirred for 5 min (800 rpm). The resulting hot emulsion (O/W) was sonicated (10 min. Subsequently, the nanoemulsion is promptly dispersed and agitated (1200 rpm) in ice bath or an aqueous PVA solution for 10 min.	SLNs prepared with γ-amino-α-hydroxy butyric acid demonstrated higher stability than SLNs of γ-aminobutyric acid. The encapsulation efficiency increased with the drug-to-lipid ratio with the highest (98%) at 1:10 (*w*/*w*). Curcumin induced apoptosis in a manner dependent on its concentration, observed in both the human breast cancer (MCF7) and the prostate cancer cell line (PC3). The cytotoxicity was additionally increased by approximately 25.4% and 34.1% with 5 μM CUR-SLN1 and CUR-SLN3, respectively.	The inclusion of the hydroxyl group in the non-polar head enhances the physical stability of SLNs, and the lipid matrix stabilizes the drug against thermal and environmental decomposition in aqueous suspension. The MTT-based cytotoxicity assay shows that SLN1 loaded with the drug significantly decreases the proliferation/viability of MCF7 and PC3 cells in a manner dependent on the dose.	[98]
Modified emulsification technique combined with sonication	Oil phase, consisting of molten lauric acid, palmitic acid, or stearic acid, was added to a hot aqueous solution comprising surfactants (Pluronic^®^ F68, Pluronic^®^ F127 (BASF, Ludwigshafen, Germany), Polysorbate 20, or Polysorbate 80 (Dae Jung Co., Ltd., Busan, Korea) and homogenized to yield an oil-in-water emulsion. Subsequently, SLNs were generated via ultrasonication using a probe sonicator at 300 W for 15 min, incorporating a 5-s pulse-on period and a 5-s pulse-off period.	Curcumin-loaded SLNs demonstrated a steady release from 7.55% to 28.63% over 48 h. Treatment with 10 μM drug loaded SLNs and pure drug solution exhibited viability in HeLa cells, CT-26 cells, and A549 cells, respectively. The viability of cells treated with drug loaded SLNs was lower compared to the drug solution, confirming the potential efficacy of the nanoparticle formulation.	The cohesive forces between curcumin and lipids include hydrogen bonding and van der Waals forces. All SLN preparations showed better anticancer effects on HeLa, A549, and CT-26 cells than the curcumin solution. A relationship between anticancer efficacy, particle size, and cell type was suggested.	[99]
Solvent evaporation	Curcumin, injectable soy lecithin, 1,2-distearoyl-sn-glycero-3-phosphoethanolamine-N-[amino (polyethylene glycol-2000)], and Compritol 888 ATO were dissolved in the ethanol phase at 70 °C. The resulting mixture was then dispersed in warm Lutrol F6 at 70 °C, followed by rapid cooling in an ice bath (2–3 °C) for 5 min. Finally, ethanol was removed under vacuum to produce drug-loaded SLNs.	Data on particle size and surface charge suggested that the physical parameters are suitable for parenteral administration. SLNs-curcumin in TgCRND8 (Tg) mice exhibited better in vivo activity (than SLNs-Tg) and was comparable with Wild Type (WT)-SLNs and WT-SLN-curcumin. In Tg-SLN mice, TG2-L expression was lower than WT-SLNs. Tg-SLNs-curcumin increased TG2-L levels, and a notable difference in Cyclin-D1 expression levels was exhibited among WT-SLNs and WT-SLNs-CUR mice.	TG2 isoforms exert effects on either the activation of apoptotic pathways or the protein’s capacity to regenerate brain cells in Tg mice. SLNs-curcumin administration in Tg mice enhanced cognitive performance and memory function, contributing to the restoration of cellular injury in these mice, thereby indicating potential therapeutic applications in Alzheimer’s disease.	[100]

**Table 4:** Summary of the preparation methods, composition, activity and key findings of curcumin-loaded NLCs.

Methods	Lipids/Surfactants	Activity	Highlights	Reference
Emulsion-evaporation- solidification	Glycerol monostearate, capric acid, lecithin, Tween 80	Wound healing and antimicrobial effect	Curcumin containing NLCs showed a potent inhibitory effect (2-fold) on gram-positive, gram-negative, and fungal organisms, exceeding curcumin’s inhibitory activity. NLCs demonstrated higher (*p* < 0.0001) wound closure compared to curcumin and the control in the first week.	[114]
Emulsion solvent evaporation	Glyceryl monostearate or lecithin, oleic acid or Labrafac, Tween 80	Antineoplastic effects	Antibody coupling and targeting efficiency were assessed by evaluating rituximab-conjugated NLCs (with curcumin and imatinib) on CD20 receptors in lymphoma cell lines. In both Jurkat T cells and Ramos B cells, the cytotoxicity resulting from co-administered drugs was higher than that of individual drugs. Co-delivery using developed NLCs holds promise for enhancing the efficacy of imatinib in managing non-Hodgkin lymphoma.	[115]
Emulsion solvent evaporation and low temperature solidification	Monostearin, octyl decyl acid triglycerate, lecithin, Poloxamer 188	Hepatocellular carcinoma	Curcumin NLCs notably elevated caspase-8 and caspase-3 activities, leading to increased apoptosis. Moreover, the increased apoptosis was suppressed in the presence of a pan-caspase inhibitor, Z-VAD-FMK. Prepared NLC triggered the activation of the extrinsic apoptosis pathway by modulating the DR5/caspase-8/-3 mediated apoptosis pathway in HepG2 cells.	[116]
Melt emulsification technique	Lysophosphatidylcholine, hydrogenated soybean oil, lecithin	Antineoplastic effects	Colon cancer cell lines, namely, HCT116 and HT29, showed enhanced uptake of curcumin compared to free curcumin when delivered using both NLC and ginsenoside-modified. Indeed, modification led to a 2-fold and 1.4-fold increase in intensity of fluorescence in HCT116 cells and HT29 cells, respectively, than plain NLC. Oral administration of modified NLC in colon cancer patients resulted in a substantial plasma level of free curcumin (~2.9 ng/mL).	[117]
Melt homogenization ultrasonication technique	Stearic acid, oleic acid, soy lecithin, Tween 20	Liver targeting	Curcumin NLCs (<200 nm), decorated with N-octadecyl-mannopyranosylamine targeting asialoglycoprotein receptors on hepatocytes, exhibited a remarkable decrease (*p* < 0.05) in serum markers of liver injury and inflammatory cytokines compared to their unconjugated counterparts.	[118]
Modified microemulsion method	Glyceryl monostearate, medium chain triglycerides, Poloxamer 188	Antineoplastic effects	NLCs loaded with temozolomide/curcumin demonstrated increased inhibitory effects on glioma cells (C6) than individual drugs. The rapid release of curcumin sensitizes cancer cells to temozolomide. There was a substantial accumulation of NLCs at brain and tumor sites, indicating a noteworthy synergistic anticancer effect without causing toxic effects on major organs and normal cells.	[119]
Nanotemplate engineering technique	Compritol® 888 ATO, oleic acid, Pluronic^®^ F68, Polysorbate 80, Span 80	Neuroprotective and antidepressant activity	Curcumin NLCs showed biphasic drug release for 1 day. Forced swim and tail suspension tests showed a significant extension of struggling time and reduction in immobility time. NLCs improved the architecture of brain tissues while decreased expression of p-NF-κB, TNF-α, and COX-2. The enhanced neuroprotective effect of curcumin suggests its potential as a therapeutic option for depression and anxiety.	[120]

**Table 5:** Composition, experimental methods, observed activity, and notable highlights of polymeric micelles loaded curcumin.

Methods	Polymers	Activity	Highlights	Reference
Dialysis	Cholesterol modified low molecular weight chitosan	Lung cancer	Successfully complexed siRNA with curcumin cholesterol-grafted chitosan micelles at N/P ratio of 40. Effective internalization of developed micelles by the human lung carcinoma A549 cell line was observed in a time-dependent manner and was confirmed using specific endocytosis inhibitors.	[139]
Dialysis	Folate-polyethylene glycol (PEG)/octadecylamine (C18)-g-polysuccinimide (PSI), PEG/ C18-g-PSI	Colon cancer	Micelles of folate-PEG/Hyd-curcumin/C18-g-PSI markedly decreased the viability of SW480 colon cancer cells in comparison to the non-folate type. Folate micelles more efficiently inhibit the Wnt/β-catenin pathway compared to others, signifying the potential for colon cancer treatment.	[140]
Solid dispersion and thin film hydration	Methoxy-poly(ethylene glycol)-block-poly(ε-caprolactone), N-(tert-butoxycarbonyl)-l-phenylalanine end-capped methoxy-poly(ethylene glycol)-block-poly(ε-caprolactone)	Erythroleukemia	The efficiency of delivering curcumin to the human pancreatic cell line (SW19990) with developed micelles was dose-dependent. Biodistribution data in rats indicated increased absorption and slower clearance of drugs in rapidly perfusing organs. A notable delay in tumor growth after intravenous administration was noticed with multidrug-resistant human erythroleukemia K562/ADR xenografts.	[141]
Solvent exchange	Poly(ethylene glycol)-*b*-poly(2-methacrylate ethyl 5-hexynoicate)	Cervical and breast cancer	The cross-linked micelles exhibited high internalization by tumor cells with lower IC_50_ against HeLa (4.86 μg/mL) and 4T1 (9.6 μg/mL) cells. These cross-linked micelles demonstrated a prolonged circulation half-life, resulting in more effective tumor inhibition compared to free drug and non-cross-linked micelles loaded with curcumin.	[142]
Sonication combined with dialysis	Poly(ethylene glycol)-crosslinked multi-armed polyethylenimine-*g*-poly(ε-benzyloxycarbonyl-L-lysine)s	Hepatic carcinoma	Curcumin-loaded micelles ensure the sustained and complete release of curcumin. Polymeric micelles amplified the rate of apoptosis in HepG2 cells, exhibited superior thermodynamic stability, heightened drug-loading capacity, improved cellular uptake, and enhanced pharmacodynamic effects in comparison to free drugs.	[143]
Thin film hydration	FLT3-specific peptide (EVQTCISHLL or EVQ)	Leukemia	Prepared micelles demonstrated remarkable internalization and enhanced curcumin accumulation in leukemic cells. They also displayed potent cytotoxic effects on MV4-11 cells while exhibiting no significant impact on normal human peripheral blood mononuclear cells.	[144]

**Table 6:** Clinical trial status for curcumin-based nanoformulations developed for diverse diseases/conditions.

Clinical Trials	Indication/Conditions	Nanocarrier	Phase	Enrolment	Identifier
Evaluate the safety, tolerability, and pharmacokinetic features of liposomal curcumin, utilizing an uncontrolled dose-escalation approach in both inpatient and outpatients at a single center. Dose administration involves intravenous infusion (8 h) once weekly for 8 weeks with dose adjustments based on the body surface area of cancer patients.	Locally advanced metastatic cancer	Liposomes	IIb	30	NCT02138955
A single-center, open-label dose-escalation study was conducted to assess the tolerance, safety, and effectiveness of liposomal curcumin intravenous infusion (3 h). This intervention is combined with radiotherapy and temozolomide in newly diagnosed persons with high-grade gliomas.	High grade gliomas	Liposomes	Phase I/II	30	NCT05768919
Assess the clinical improvement in amyotrophic lateral sclerosis patients with the combined treatment of liposome encapsulated curcumin and resveratrol (G04CB02).	Amyotrophic lateral sclerosis	Liposomes	Phase II	90	NCT04654689
Investigate the impact of nanoemulsion curcumin on pro-inflammatory biomarkers in plasma and breast adipose tissue. Assess the adherence, tolerability, and safety of two doses of nanoemulsion curcumin in women at a high risk of developing breast cancer.	Breast cancer	Nanoemulsion	Interventional	29	NCT01975363
A randomized, double blinded pilot study to assess the feasibility of employing functional assessment of cancer therapy endocrine symptoms scores for detecting alterations in symptoms and well-being induced by aromatase inhibitors in postmenopausal women with breast cancer after 3 months of nanoemulsion curcumin compared to placebo.	Breast cancer	Nanoemulsion	Phase I	42	NCT03865992
Investigate the impact of simultaneously administering curcumin and micelle entrapped curcumin, both individually and in combination, to enhance oral bioavailability. Additionally, assesses potential age and sex-related differences in curcumin pharmacokinetics.	Safety and Pharmacokinetics of new curcumin formulations	Polymeric micelles	Phase I	23	NCT01982734
Examine the impact of micellar curcumin on markers related to inflammation and lipid metabolism in individuals at risk for metabolic syndrome.	Safeguarding against metabolic syndrome	Polymeric micelles	Phase II	42	NCT01925547
Assess the clinical and radiographic outcomes of using nano propolis and nano curcumin as direct pulp capping agents in young permanent teeth.	Traumatic pulp exposure in children	Nanoparticles	Phase I	54	NCT06029023
Assess the clinical effectiveness of a novel nano-technology-based topical curcumin gel and compare it with a 2% curcumin gel in patients experiencing recurrent aphthous ulcers.	Recurrent aphthous ulcer and stomatitis	Nanoparticles	Interventional	48	NCT04385979
Evaluate the influence of curcumin nanoparticles on improving behavioral measures and biomarkers associated with cognition and neuroplasticity in individuals with schizophrenia who are presently on a stable dose of antipsychotic medication.	Schizophrenia	Nanoparticles	Phase I and II	39	NCT02104752

**Table 7:** A compilation of recently filed patents for curcuminoids and highlights of their innovation.

Application ID	Title	Summary of Invention	Date of Publication
US 20230310536 A1	Water soluble curcuminoid composition for treating mouth and throat conditions	Formulation designed for oral, tongue and throat medical conditions consists of curcuminoids solubilized by aqueous medium or the hydrophilic carrier.	2023-10-05
US 20230293696 A1	Curcuminoid composites	A melted composite containing curcuminoids and a carrier chosen from rice bran extract, mannitol, maltodextrin, or their mixtures. These composites, formulated in solid, liquid, or semisolid compositions, demonstrate rapid dissolution and improved bioavailability.	2023-09-21
US 20230225992 A1	Liquid dispersible curcuminoid compositions and methods of improving cognitive function.	The method involves preparing a composition by combining curcuminoid or its derivative with a dispersing agent under high shear. This composition elevates serum concentrations of brain-derived neurotrophic factors, potentially improving cognitive function, even when co-administered with iron.	2023-07-20
US 20230190856 A1	A composition comprising complex comprising curcuminoid compound, and steviol glycosides or a licorice extract or a fraction thereof, and uses thereof.	A formulation consisting of a complex containing a curcuminoid-based compound and a steviol glycoside, along with a licorice extract or a fraction, is designed to alleviate symptoms of COVID-19 by stimulating Th1 cells, CD8 T cells, and NK cells.	2023-06-22
US 20220280449 A1	Curcuminoid compositions	Methods and formulations containing highly water soluble curcuminoids utilized for various applications.	2022-09-08
US 20220280448 A1	Uses of curcuminoid compositions	Methods and applications involve compositions with curcuminoids to enhance bioaccessibility, bioavailability, bioefficacy, and/or bioactivity in mammals.	2022-09-08
US 20220184170 A1	Formulation to enhance the bioavailability and stability of curcuminoids and/or its derivatives thereof	A formulation containing turmeric extract showcases improved aqueous solubility and bioavailability, with additional components such as piperine, turmeric oil, acrysol K-140 and beeswax.	2022-06-16
US 20220184001 A1	Therapeutically administrable high dose non-aqueous curcuminoid solutions	Curcuminoid in glycerol, along with sufficient alkali salt in specific embodiments, enhances solubility, suitable for direct intramuscular delivery, and remains stable for over 24 h without curcuminoid precipitation.	2022-06-16
US 20220125931 A1	Pharmaceutical preparation with curcuminoids nanoparticles and a method for producing the same	The composition contains curcuminoids (4–8%), oil phase (20–35%), co-solvent (25–35%) and surfactant (40–50%). The purified curcuminoids (~50%) exhibit an average size of approximately 19 nm.	2022-04-28
US 20220054580 A1	Formulation comprising water soluble particles of a non-curcuminoid and a curcuminoid	The formulation contains either turmeric oleoresin, curcumin-extracted turmeric oleoresin, turmeric oil, or a combination of these. The product is water soluble without adding any buffer and also includes a curcuminoid.	2022-02-24
US 20210330591 A1	Curcuminoid composites	A melt composite, consisting of one or more curcuminoids and a carrier chosen from rice bran extract, mannitol, maltodextrin, and their mixtures. The formulation can exist in solid, liquid, or semisolid compositions, all demonstrating rapid dissolution and improved bioavailability.	2021-10-28

## Data Availability

The data presented in this study is contained within this article.
